# Local euchromatin enrichment in lamina-associated domains anticipates their repositioning in the adipogenic lineage

**DOI:** 10.1186/s13059-022-02662-6

**Published:** 2022-04-11

**Authors:** Julia Madsen-Østerbye, Mohamed Abdelhalim, Marie-Odile Baudement, Philippe Collas

**Affiliations:** 1 Department of Molecular Medicine, Institute of Basic Medical Sciences, Faculty of Medicine, University of Oslo, 0317 Oslo, Norway; 2 Present Address: Centre for Integrative Genetics, Faculty of Biosciences, Norwegian University of Life Sciences, 1430 Ås, Norway; 3 Department of Immunology and Transfusion Medicine, Oslo University Hospital, 0424 Oslo, Norway

**Keywords:** Adipose stem cell, Adipogenesis, Enhancer, H3K27 acetylation, LAD, Lamina-associated domain

## Abstract

**Background:**

Interactions of chromatin with the nuclear lamina via lamina-associated domains (LADs) confer structural stability to the genome. The dynamics of positioning of LADs during differentiation, and how LADs impinge on developmental gene expression, remains, however, elusive.

**Results:**

We examined changes in the association of lamin B1 with the genome in the first 72 h of differentiation of adipose stem cells into adipocytes. We demonstrate a repositioning of entire stand-alone LADs and of LAD edges as a prominent nuclear structural feature of early adipogenesis. Whereas adipogenic genes are released from LADs, LADs sequester downregulated or repressed genes irrelevant for the adipose lineage. However, LAD repositioning only partly concurs with gene expression changes. Differentially expressed genes in LADs, including LADs conserved throughout differentiation, reside in local euchromatic and lamin-depleted sub-domains. In these sub-domains, pre-differentiation histone modification profiles correlate with the LAD versus inter-LAD outcome of these genes during adipogenic commitment. Lastly, we link differentially expressed genes in LADs to short-range enhancers which overall co-partition with these genes in LADs versus inter-LADs during differentiation.

**Conclusions:**

We conclude that LADs are predictable structural features of adipose nuclear architecture that restrain non-adipogenic genes in a repressive environment.

**Supplementary Information:**

The online version contains supplementary material available at 10.1186/s13059-022-02662-6.

## Background

High-order spatial genome organization involves chromatin compartmentalization, interactions between topological domains, and associations of chromatin with the nuclear lamina, a meshwork of A- and B-type lamins (lamin A/C, lamin B1 and lamin B2) at the nuclear envelope [1]. Lamina-associated domains (LADs) are typically heterochromatic (enriched in H3K9me2 and H3K9me3) and harbor a low density of genes that are mostly repressed or expressed at low level [2]. LADs occupy 30–40% of the genome and are largely conserved between cell types [3, 4], leading to the notion of constitutive LADs (cLADs) being a structural feature of nuclear architecture, segregating repressed parts of the genome to the nuclear periphery.

Some LADs however, classified as facultative or variable LADs (vLADs), differ between cell types [3, 4] and arise during differentiation [5–8], during the cell cycle [9], or across circadian time [10]. Whether vLADs occur via regulated or stochastic lamin-genome interactions is however unclear. The significance of vLADs also remains obscure because they do not strongly correlate with changes in gene expression [1]. This raises the question of how genes in vLADs are regulated.

vLADs have mostly been characterized without distinction of whether they occur as stand-alone LADs—that is, entire LADs which emerge or disappear, or as variations in the length of existing LADs. Being able to distinguish between these features is important in order to be able to interpret gene expression changes in these domains. Indeed, genes located in edges of LADs are potentially conflicted by the chromatin environment of the LAD itself and of the adjacent inter-LAD (i-LAD). On the other hand, LAD edges may act as structural modulators of transcription by releasing enhancers that can regulate the activity of neighboring genes [11, 12].

Developmentally regulated loci also lose interactions with A- or B-type lamins during adipogenic differentiation [8, 13]. However, whereas release of genes from the nuclear lamina may coincide with enhanced expression [8], many genes are transcriptionally oblivious to a change in LAD status [6]. Accordingly, over 10% of genes in LADs are expressed [2] while others remain silent even when experimentally introduced into an i-LAD environment [14]. This discordance has been explained by promoter sequence characteristics and variations in histone post-translational modifications (PTMs) in LADs [14–16]. Whether developmentally regulated genes can escape repressive LAD environments remains, however, unknown.

Adipose stem cells (ASCs) isolated from human adipose tissue constitute a valuable ex vivo system to study cellular and gene regulatory changes taking place during adipogenic differentiation [6, 17–21]. Following induction of human or mouse ASC differentiation into adipocytes, multiple waves of transcriptional up- and downregulation driving adipogenesis [20, 21] are accompanied by changes in 3-dimensional chromatin organization involving long-range associations between topological chromatin domains [22], lineage-specific repositioning of lamin A/C on promoters [13], acquisition of new chromatin states [6, 21], and a remodeling of transcription factor networks [23], chromatin accessibility [24], and promoter-enhancer and enhancer-enhancer interactions [25, 26]. To our knowledge however, there are no reports on changes in transcriptomic and histone PTMs over short time periods after initiation of adipogenic differentiation, and on the interplay between such changes and a repositioning of LADs.

Here, we examined the temporal associations of lamin B1 (LMNB1) with the genome in relation to differential gene expression during the first 72 h of adipogenic differentiation. We show a non-stochastic repositioning of stand-alone LADs and LAD edges which is either concordant or discordant with changes in gene expression in these domains. Differentially expressed genes within LADs reside in local euchromatic and lamin-depleted sub-domains, where promoter and enhancer histone PTMs before differentiation forecast the LAD vs. i-LAD outcome of these genes during differentiation. We conclude that LADs emerge as determinative structural features of adipose nuclear architecture.

## Results

**Fig. 1:**
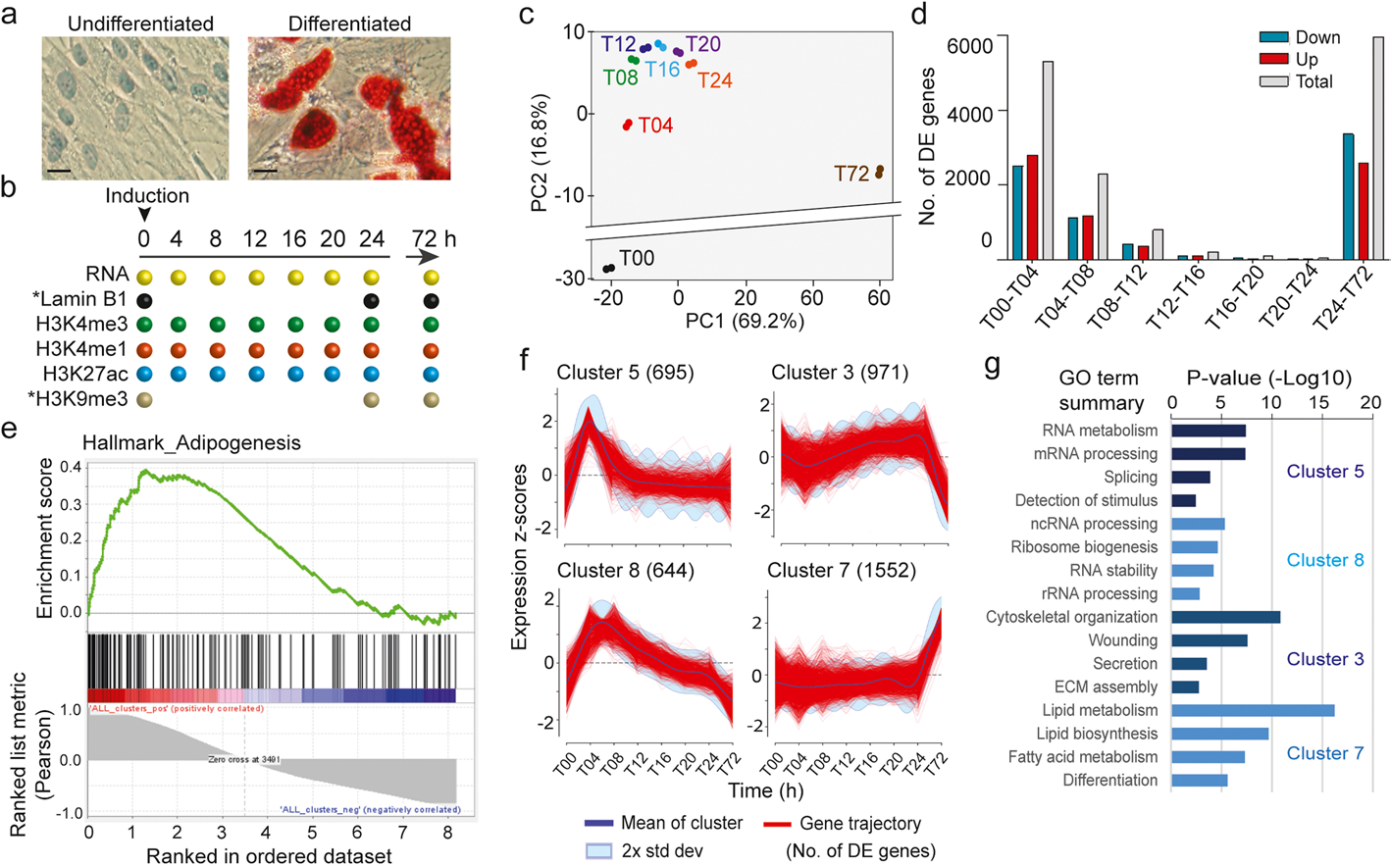
Adipogenic induction elicits sequential waves of transcriptional up- and downregulation. **a** Assessment of ASC differentiation into adipocytes by Oil Red-O staining of intracellular lipid droplets in undifferentiated ASCs and in day 15 adipocytes. Scale bars, 20 μm. **b** Time points of data collection for this study. *Re-analyzed published datasets from Ref. [22]. **c** Principal component analysis of gene expression from 0 to 72 h in each differentiation replicate (% of variance). **d** Numbers of differentially expressed genes (DE; FDR ≤ 0.05) between two consecutive differentiation time points. **e** GSEA of differentiating ASCs (0–72-h time course); Hallmark_Adipogenesis. **f** Unsupervised expression clusters of DE protein-coding genes based on their mean expression *z*-scores (numbers of genes shown); see Additional file 1, Fig. S2b for all clusters. **g** Gene ontology terms (PANTHER biological process) enriched in the expression clusters shown in **f**; Fisher’s exact test adjusted *P*-values with FDR ≤ 0.05

Our system to investigate the dynamics of nuclear lamina-chromatin interactions consists in the differentiation of human primary ASCs into adipocytes (verified by Oil Red-O staining of intracellular lipids; Fig. 1a), which we have previously characterized at the phenotypic, transcriptional and epigenetic levels [6, 13, 17, 19, 21]. Here, we focus on the first 72 h of differentiation to examine the dynamics of early LAD repositioning during initiation of the adipogenic gene expression program. A flow cytometry-based carboxyfluorescein succinimidyl ester intracellular dye dilution assay establishes that ASCs do not divide at the onset of differentiation (time 0 h; T00) and thereafter, and Western blotting analysis indicates that total LMNB1 protein levels are comparable during this differentiation time course (Additional file 1, Fig. S1a, b). Thus, our ASC system provides a useful setting to study the repositioning of LADs during differentiation without confounding effects of varying lamin protein levels and of LAD reformation after disassembly and reassembly of the nuclear envelope [27].

In two independent experiments, proliferating ASCs were seeded at confluency and induced 48 h later with an adipogenic cocktail [17]. Cells were harvested immediately before induction (T00), every 4 h over the first 24 h (T04-T24) and at 72 h (T72) for transcriptome analysis by RNA-seq and analysis of histone modifications by ChIP-seq (Fig. 1b). RNA-seq data confirm the induction of differentiation by the downregulation of ASC stemness genes and the upregulation of early adipogenic genes (Additional file 1, Fig. S2a). Principal component analysis reveals marked transcriptomic changes as early as 4 h (T04; Fig. 1c), when we also detect the highest numbers of significantly differentially expressed genes (DE; FDR ≤ 0.05; Fig. 1d). Gene set enrichment analysis (GSEA) of all 8175 DE protein-coding genes over the 72-h time course against the Hallmark “Adipogenesis” indicates that the upregulated genes positively correlate with gene sets displaying an adipogenic signature (Fig. 1e). Moreover, unsupervised expression clustering of DE genes in the time course reveals sequential waves of transcriptional up- and downregulation, notably at 4 h (cluster 5), 4–8 h (cluster 8), 24 h (cluster 3), and 72 h (cluster 7) (Fig. 1f; Additional file 1, Fig. S2b; see Additional file 2, Table S1 for gene lists in each cluster).

To gain insights into transcriptional pathways triggered by differentiation, we queried Gene Ontology (GO) terms enriched in the most abundant up- and downregulated gene clusters (Fig. 1g). GO analysis highlights sequential and transient upregulation of genes involved in stimulus detection, RNA metabolism, and ribosome biogenesis in the first 4–8 h (cluster 5, 8), reflecting the initiation of transcription and translation programs, followed by cytoskeleton rearrangement and extracellular matrix organization by 12–24 h (cluster 3), and from 24 h onwards, lipid biosynthesis and fatty acid metabolism, hallmarks of adipogenesis (cluster 7; Fig. 1g). This demonstrates the activation of an immediate/early transcriptional program promoting engagement of ASCs into the adipogenic lineage.

### Tracking LADs after induction of adipogenic differentiation

**Fig. 2:**
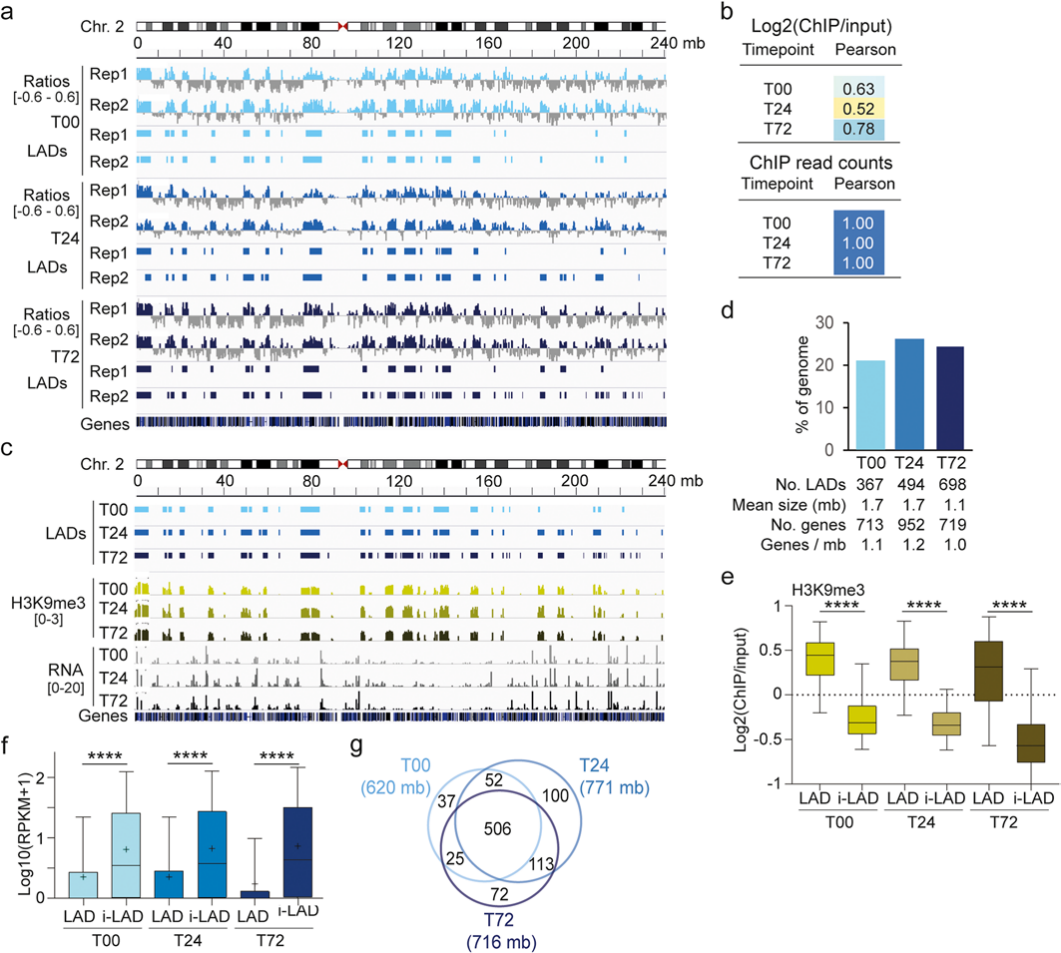
LADs are dynamic features of adipogenic differentiation. **a** Genome browser view of LMNB1 Log2(ChIP/input) ratios (*y* axis range shown in brackets) and called LADs for each differentiation replicate (Rep1, Rep2) at T00, T24, and T72. Other examples are shown in Additional file 1, Fig. S3. **b** Pearson correlations of Log2(LMNB1 ChIP/input) ratios across the genome and of LMNB1 ChIP read counts in mapped LADs, between differentiation replicates. **c** Genome browser view of LADs, H3K9me3 enrichment (Log2(ChIP/input) ratio range shown in brackets), and mRNA level (read count scale shown in brackets) in the 0–72-h time course. **d** Percent of genome coverage and genomic properties of LADs at T00, T24, and T72. **e** H3K9me3 enrichment in LADs and i-LADs; bar, median; box, 25–75% percentile; whiskers, min-max; ****P < 10^−4^, ANOVA with Welch’s correction. **f** Expression level of all protein-coding genes in LADs and i-LADs; cross, mean; bar, median; box, 25–75% percentile; whiskers, min-max; ****P < 10^−4^, ANOVA with Welch’s correction. **g** Venn diagram of genome coverage by LADs at each time point

To investigate the relationship between initiation of this adipogenic program and genome architecture, we mapped LADs during the 72-h time course by re-analyzing our LMNB1 ChIP-seq data from a recent duplicate adipose differentiation with the same batch of ASCs as that used in this study [22] (Fig. 1b). We examined LMNB1 enrichment profiles and called LADs in each differentiation replicate using Enriched Domain Detector. Pearson correlations of LMNB1 enrichment (Log2(ChIP/Input)) in 100-kilobase (kb) bins across the genome (Fig. 2a; Additional file 1, Fig. S3a, b) and of ChIP read counts in the called LADs both denote high reproducibility between differentiation replicates (Fig. 2b). On this basis, we identified, from domains merged between replicates, 367–698 LADs in the time course (Fig. 2c). As expected, these LADs have low gene density (Fig. 2d), are enriched in H3K9me3 relative to i-LADs (Fig. 2e), and overall display low gene expression levels (Fig. 2f). Intersection of LADs at each differentiation time point reveals 506 megabase (mb) of cLADs, as a structural feature of ASCs and early adipocytes, and vLADs, some being unique to each time point (Fig. 2g).

**Fig. 3:**
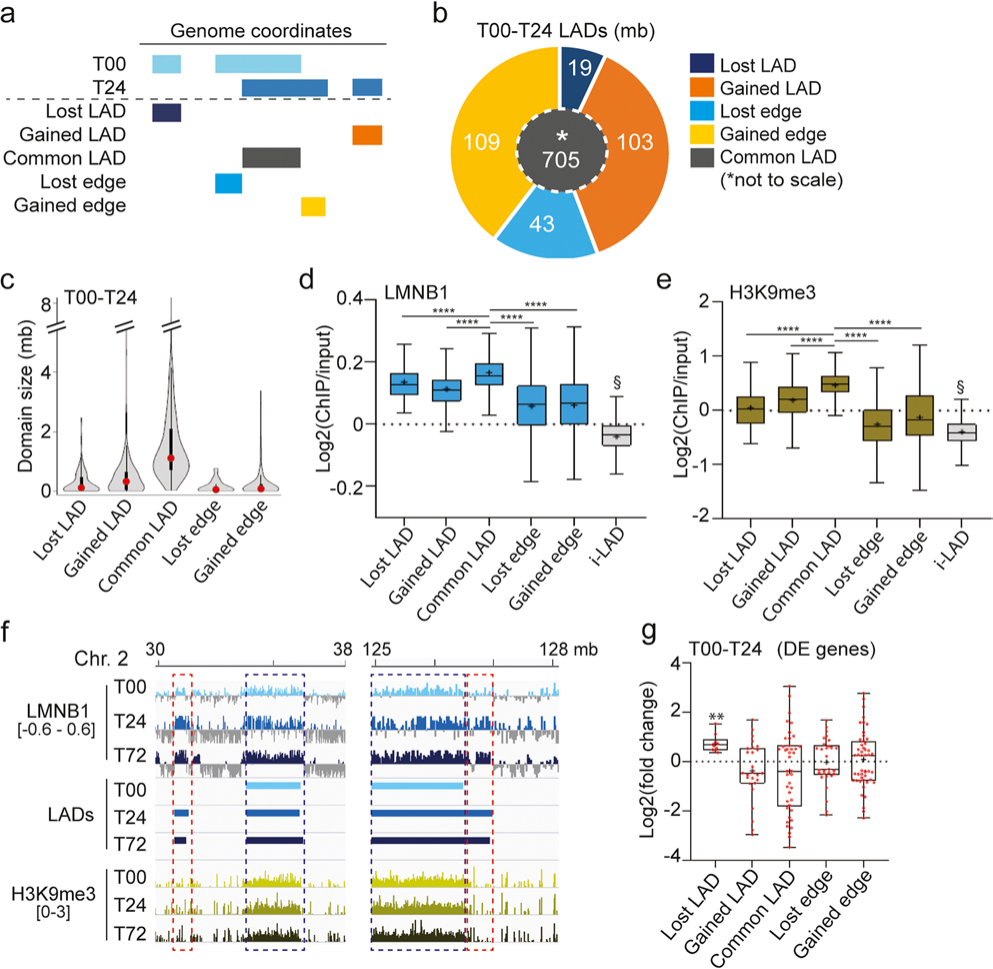
Maintenance, loss, and gain of LADs in the first 24 h of adipogenic differentiation. **a** Definition of lost/gain LADs and LAD edges. **b** Proportions of common LADs (center), gained/lost LADs, and gained/lost LAD edges between T00 and T24 (mb coverage). **c** Violin and box plot of LAD size distribution per LAD category; box plot: dot, median; box: 25–75% percentile; whiskers, min-max. **d** LMNB1 and **e** H3K9me3 enrichment in each LAD category and i-LADs in the 0–24-h time course; ****P < 10^−4^, and ^§^*P* < 10^−4^ relative to all LAD category; ANOVA with Welch’s correction. **f** Genome browser views of Log2(ChIP/input) ratios for LMNB1 and H3K9me3 (*y* axis range shown in brackets) in vLADs (red boxes) and cLADs (blue boxes). **g** Fold-change of expression of DE genes between 0 and 24 h in each T00–T24 LAD category; bar, median; cross, mean; box, 25–75% percentile; whiskers, min-max; dots, data points. **P = 0.002 (one-sample *t*-tests)

To characterize the positioning of these vLADs during differentiation, we tracked the fate of LADs of undifferentiated T00 ASCs (T00 LADs) over the 72-h time course. We first identify a 212-mb gain of stand-alone LADs and LAD edges between 0 and 24 h, and a less prominent loss of LADs and LAD edges (62 mb; Fig. 3a, b; Additional file 1, Table S2). Gained or lost LADs or edges are smaller than common LADs (Fig. 3c), suggesting that they are prone to less stable interactions with the nuclear lamina. Accordingly, vLADs harbor significantly lower LMNB1 and H3K9me3 enrichment than common LADs (*P* < 10^−4^, ANOVA with Welch’s correction; Fig. 3d–f). Moreover, DE genes in the 0–24-h time course, which are localized in LADs, are or tend to be up- and downregulated in lost and gained LADs, respectively; this is not significant in gained and lost edges, likely due their proximity to common LADs (Fig. 3g). Thus, both gains and losses of whole LADs and extension/shortening of LAD edges are features of chromatin reorganization during early adipogenesis.

**Fig. 4:**
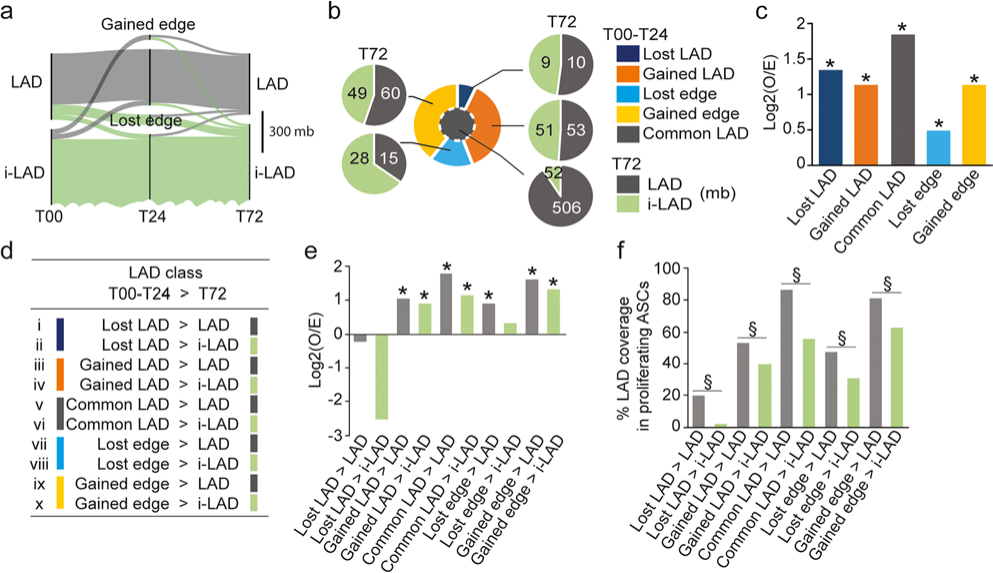
LAD fate in the 72-h differentiation time course. **a** Alluvial representation of LAD fate. Line width is proportional to genomic size. **b** Proportions of each T00–T24 LAD category (center pie chart; see Fig. 3b) in LAD (gray) or in i-LAD (green) at T72 (mb coverage). **c** Observed/ expected (O/E) ratios of genomic overlap of T00–T24 LAD categories (*x* axis) with LADs at 72 h. Each LAD was permuted 50 times in the same chromosome using as background the union of all known LAD locations in T72 cells; *P = 0.019, two-sided permutation tests, FDR ≤ 0.1, for all LAD categories; see also Additional file 1, Table S3. **d** LAD classes identified based on LAD changes between 0 and 24 h (T00–T24) and the LADs vs. i-LAD outcome of these LADs by 72 h (T72). **e** Observed/expected (O/E) ratios of overlap of each LAD class (*x* axis) with LADs in undifferentiated proliferating ASCs. *P = two-sided permutation tests (250 permutations), FDR ≤ 0.1. See also Additional file 1, Table S4. **f** Proportions of genome coverage of LADs in undifferentiated proliferating ASCs by each LAD class; ^§^*P* < 0.002, Fisher’s exact tests with two-tailed *P*-values

To examine this LAD dynamics more closely, we tracked the LAD vs. i-LAD fate of each T00-T24 LAD change at 72 h (T72). Alluvial representation and quantification of LAD changes indicate that while T00–T24 LADs largely remain LADs by 72 h (defining cLADs), vLADs overall segregate in similar proportions into LADs and i-LADs (Fig. 4a, b). To determine whether this reflected a preferred LAD fate, we tested the null-hypothesis of a random segregation of these LADs in LAD regions at 72 h. We find that overlaps of these LADs with T72 LADs are greater than what would be expected from a random distribution (*P* = 0.019, two-sided permutation tests, FDR ≤ 0.1; Fig. 4c; Additional file 1, Table S3), suggesting that the fate of 0–24 h LADs and i-LADs is non-random. From these analyses, we identify ten LAD classes summarizing the fate of ASC LADs in the first 72 h of adipose differentiation (Fig. 4d; Additional file 1, Table S2 and Fig. S4a).

### LADs in undifferentiated ASCs are maintained or recovered post-differentiation

While cLADs appear as a conserved feature of chromatin organization in ASCs, we do not know whether during early adipogenesis, vLADs emerge in regions that are pre-determined in ASCs regardless of any synchronization or differentiation. To address this, we examined whether the post-differentiation LAD distribution observed by 72 h (see Fig. 4b) could be traced back in proliferating ASCs. To this end, we computed O/E ratios of overlap of each LAD class with LADs previously mapped by us in proliferating ASCs [22] (Additional file 1, Table S4). LADs in all classes, except “Lost LADs,” have a higher propensity to be “in LADs” in proliferating ASCs than what would be expected from a random distribution (Fig. 4e; *P = 0.004, two-sided permutation test, FDR ≤ 0.1). In addition, domains with a T72 LAD outcome overlap more significantly with LADs in proliferating ASCs than those with an i-LAD outcome (Fig. 4f; ^§^*P* < 0.002; Fisher’s tests with two-tailed *P*-values). These results indicate a prior non-random enrichment of T72 LADs in LAD domains established in proliferating ASCs. This suggests a view of memory of LAD positioning in undifferentiated ASCs, which is stored during differentiation induction and recalled by 72 h when cells engage in adipogenesis.

### vLADs are associated with gene ontologies consistent with induction of differentiation

**Fig. 5:**
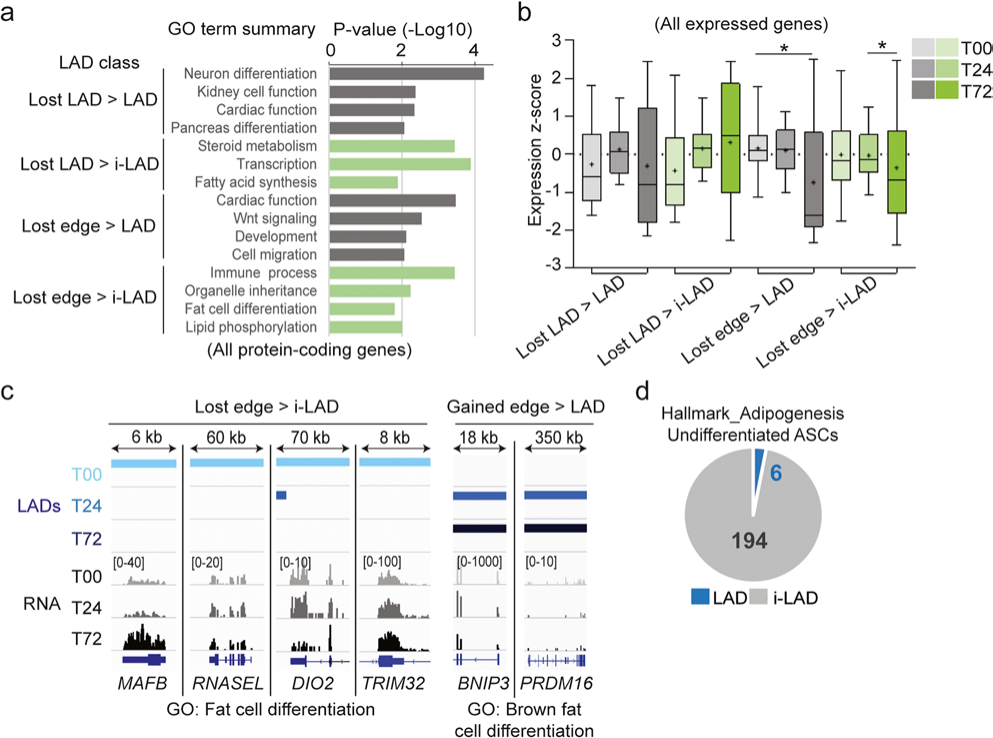
vLADs are associated with gene ontologies consistent with induction of adipogenic differentiation. **a** Gene ontology terms (PANTHER biological process) associated with all protein-coding genes found in “Lost LAD” and “Lost edge” LAD classes. See Additional file 1, Table S6 for GO term summaries in all LAD classes. **b** Expression *z*-scores of all expressed protein-coding genes in LAD classes shown in **a**, at T00, T24, and T72. Bar, median; cross, mean; box, 25–75 percentile, whiskers min-max; *P = 0.04, unpaired two-tailed *t*-test with Welch’s correction. **c** Genome browser views of LADs and mRNA levels (read count scales shown in brackets) for indicated DE genes; note that LADs extend beyond the areas shown. **d** Numbers of adipogenic genes (GSEA Hallmark_Adipogenesis) in LADs and i-LADs in undifferentiated (T00) ASCs

To gain insight into the functionality of the differentiation-induced vLADs, we queried Gene Ontology (GO) terms enriched for genes uniquely found in each LAD class irrespectively of whether they are expressed or not (see Additional file 2, Table S5 for gene lists in each LAD class). GO analysis (Additional file 1, Table S6) reveals that genes characterizing non-adipogenic lineages (e.g., neuronal, nephric, cardiac, pancreas) are confined into LADs at 72 h irrespective of their prior LAD localization (Fig. 5a; > LAD classes). In contrast, we find genes involved in fatty acid synthesis and white adipocyte differentiation and function in lost LADs or edges by 72 h, also regardless of their prior LAD localization, reflecting their release into a permissive environment (Fig. 5a; > i-LAD classes). Moreover, while most genes in each LAD class are repressed, 11–57% are expressed at least one time point (Additional file 1, Table S2); some of these genes are also up- or downregulated (significantly or as a trend) in a direction that agrees with a loss or gain of LAD (Fig. 5b; Additional file 1, Fig. S4b). As examples, the adipogenic genes *MAFB*, *RNASEL*, *DIO2*, or *TRIM32* are released from LAD edges between 0 and 24 h and upregulated by 24 or 72 h (Fig. 5c, left panel). Anecdotally, two genes involved in brown adipogenesis, *BNIP3* (downregulated) and *PRMD16* (not expressed), are in gained edges by 24 h which remain LADs (Fig. 5c, right panel), consistent with the distinct developmental lineage of white and brown adipocytes [28].

These results indicate that vLADs compartmentalize genes involved in non-adipogenic lineages in repressed parts of the genome and may contribute to reinforcing the repression of loci irrelevant for adipogenesis. In parallel, genes important for adipocyte function lose lamin association. Note however that the majority of adipogenic genes is already outside LADs in undifferentiated ASCs (Fig. 5d), in line with the lineage commitment of these cells. Thus, whereas release of adipogenic genes from LADs in our system is not a dominant characteristic of adipose differentiation, sequestration or maintenance of non-adipogenic genes in LADs is an enhanced feature.

### Constitutive and variable LADs host differentially expressed genes

**Fig. 6:**
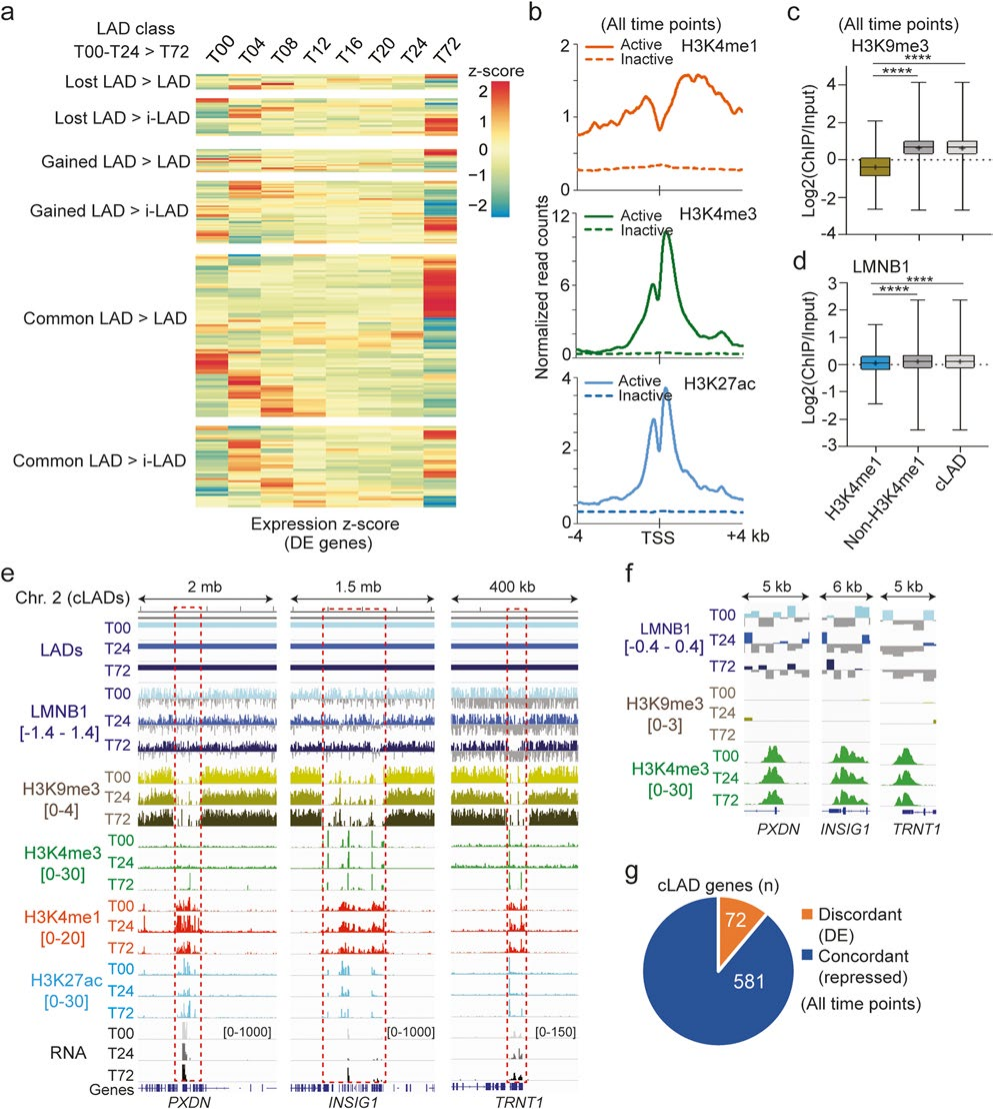
Active genes in each LAD class are localized in euchromatic environments of low lamin enrichment. **a** Mean expression *z*-score of DE genes in each LAD class, scaled across time points; data are also shown with gene names for all LAD classes in Additional file 1, Fig. S5. **b** H3K4me1, H3K4me3, and H3K27ac levels around the TSS of all expressed (active; *n* = 116) and non-expressed (inactive; *n* = 3702) genes in cLADs (mean ChIP-seq read counts normalized to library size and averaged across all 0–72-h time points). An expressed gene is a gene with a normalized read count ≥ 15 at at least one time point. **c** H3K9me3 enrichment and **d** LMNB1 enrichment in H3K4me1 regions and in non-H3K4me1 regions in cLADs, and across the entire cLADs; bar, median; cross, mean; box, 25–75% percentile; whiskers, min-max ****P < 10^−4^, ANOVA with Welch’s correction. An H3K4me1 region is a cLAD region containing at least one H3K4me1 peak at at least one time point in the 0–72-h time course. A non-H3K4me1 region never contains any H3K4me1 peak at any time point. **e** Genome browser views of reduced LMNB1 and H3K9me3 enrichment at DE genes in cLADs (boxed areas). Enrichments of H3K4me3, H3K4me1, and H3K27ac are shown; ranges of Log2(ChIP/input) ratios shown in brackets; mRNA levels shown with read count scales in brackets. **f** Zoom-in views of LMNB1 and H3K9me3 depletion at the *PXDN*, *INSIG1*, and *TRNT1* start sites; H3K4me3 enrichments are also shown; ranges shown in brackets. **g** Numbers of repressed genes (concordant with the overall repressed status of LADs) and of DE genes (discordant with LAD status) in cLADs, all time points confounded

To provide functionality to LAD changes elicited by differentiation, we examined more closely the temporal changes in expression of DE genes in each LAD class. We find that in all classes, including cLADs, a number of these genes show evidence of upregulation around T04–T08 and follow distinct expression profiles over time thereafter (Fig. 6a; Additional file 1, Fig. S5a-d). Moreover, many expression profiles surprisingly do not concur with what would be expected from their LAD status (Fig. 6a), indicating that DE genes experience transcriptional changes that are uncoupled from their LAD context.

The resilience of DE genes to changes in LAD status suggests that they may be under the influence of local chromatin states distinct from the heterochromatic LAD environment. To assess this, we profiled by ChIP-seq H3K4me3, marking promoters of active genes, H3K27ac, marking active enhancers and promoters, and the enhancer mark H3K4me1, every 4 h between T00 and T24. Total levels of these histone PTMs, assessed by Western blotting, did not significantly vary over time (Additional file 1, Fig. S6a-c). We find that these PTMs are enriched around transcription start sites (TSSs) in a manner consistent with promoter activity in cLADs at ≥ 1 time point in the 0–24-time course (Fig. 6b). In addition, H3K4me1 regions in cLADs are depleted of H3K9me3 (Fig. 6c) and exhibit lower LMNB1 enrichment than non-H3K4me1 regions (*P* < 10^−4^, ANOVA with Welch’s correction; Fig. 6d). Closer examination of DE genes important for adipogenesis (*PDXN*, *INSIG1*, or *TRNT1*) confirms the absence of H3K9me3, enrichment in H3K4me1, H3K4me3, and H3K27ac, and the local depletion of LMNB1 at the TSS (Fig. 6e, f). DE genes discordant with LAD association make up 11% of all protein-coding genes in cLADs (Fig. 6g). We conclude that DE genes in cLADs display reduced LMNB1-promoter contact frequencies and locally evade the overall heterochromatic environment of cLADs.

### Histone modifications in pre-differentiation LADs correlate with post-differentiation LAD outcome

**Fig. 7:**
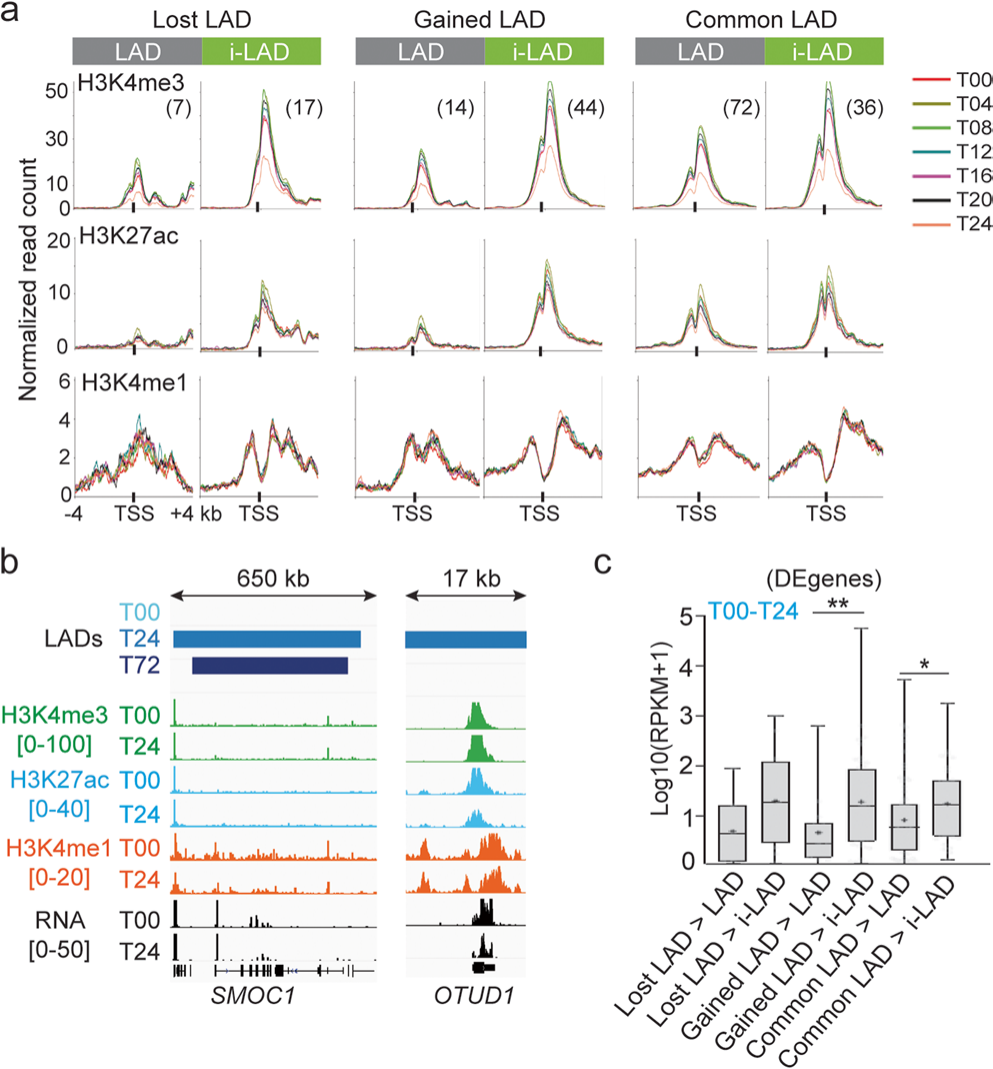
Heterogeneity in histone modifications and gene expression in LADs. **a** Aggregation plots of H3K4me3, H3K27ac, and H3K4me1 levels around the TSS of DE genes in indicated LAD classes (top) in the T00–T24 time course (numbers of DE genes); mean ChIP-seq read counts normalized to library size at each time point. See Additional file 1, Fig. S7a for LAD edges. **b** Genome browser views of LADs, H3K4me3, H3K27ac, and H3K4me1 enrichment (range Log2(ChIP/input) ratios shown in brackets), and of RNA levels (read count scale in brackets) at the *SMOC1* (gained LAD > LAD) and *OTUD1* locus (gained LAD > i-LAD). Note that LADs at T24 extend beyond the area shown. **c** Expression levels of DE genes in indicated LAD classes averaged across the T00–T24 time course; bar, median; cross, mean; box, 25–75% percentile; whiskers, min-max. *P = 0.03, **P = 0.01, unpaired two-tailed *t*-tests with Welch’s correction

Since we previously identified promoter histone PTM profiles explaining the discordance of DE gene expression in cLADs, we next determined the temporal relationship between these PTMs and LAD fate in other LAD classes. Strikingly, H3K4me3, H3K27ac, and H3K4me1 signal strengths in both lost/gained LADs and cLADs are distinct at the TSS of genes subsequently destined to be in i-LADs from those confined to LADs (Fig. 7a). As examples, the *SMOC1* and *OUTD1* genes gain a LAD by 24 h and respectively retain it or lose it by 72 h (Fig. 7b). This is not observed in LAD edges, probably due to conflicting chromatin states at LAD/i-LAD interfaces (Additional file 1, Fig. S7a). Thus, promoter H3K4me1/me3 and H3K27ac in LADs in the first 24 h of differentiation correlates with ensuing LAD outcome, but intriguingly not with the LAD versus i-LAD context at 0 or 24 h.

We thus asked whether this differential histone PTM marking was related to gene expression level, which also varies in LADs (see Fig. 6a). Indeed, histone PTMs also correlate with stronger expression of vLAD genes (except in edges) destined to i-LADs as opposed to LADs (Fig. 7c; Additional file 1, Fig. S7b). We conclude that in stand-alone vLADs and to some extent in cLADs, both promoter histone PTMs and gene expression in the 0–24-h time frame anticipate subsequent LAD outcome. Moreover, and importantly, H3K4me3, H3K4me1, and H3K27ac profiles are all established before differentiation onset (T00; Fig. 7a), raising a view of epigenetic pre-patterning, in undifferentiated cells, of differential gene expression and LAD outcome post-differentiation.

### Co-segregation of enhancers imputed to differentially expressed LAD genes during differentiation

**Fig. 8:**
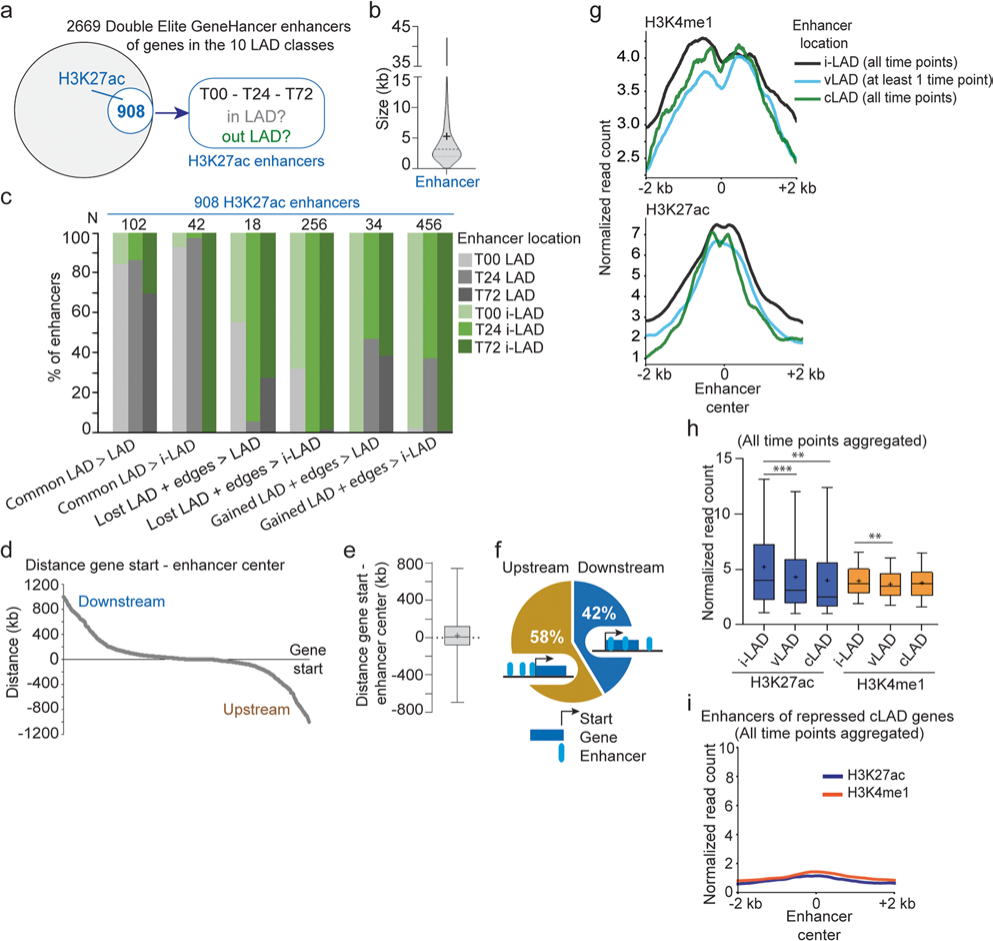
Co-segregation of enhancers with target genes in vLADs or cLADs. **a** Identification of H3K27ac enhancers used in this study. **b** Size distribution of the GeneHancer double-elite enhancers considered in this study; thick bar, median; thin bars, 25–75% percentile; cross, mean. **c** Proportions of H3K27ac enhancers in LADs (gray shades) or i-LADs (green shades) at T00, T24, and T72, which are targeted to genes localized in indicated LAD classes (*x* axis); see Additional file 2, Table S7 for numbers of enhancers localized in each LAD class. **d** Distribution of gene start-enhancer distances for enhancers targeted to DE genes in all LAD classes; distances are from enhancer center to gene start sites, upstream (negative values) or downstream (positive values) of the gene start sites. **e** Box plot of gene start-enhancer center distances; bar, median; cross, mean; box, 25–75% percentile; whiskers, min-max. **f** Proportions of upstream and downstream enhancers. **g** H3K4me1 and H3K27ac levels at enhancers located in i-LADs, vLADs, and cLADs (all time points aggregated). vLAD enhancers are defined as enhancers in a vLAD at ≥ 1 time point in the 0–72-h time course; cLAD enhancers are in cLADs at all time points; i-LAD enhancers are in i-LADs at all time points; mean ChIP-seq read counts normalized to library size across all time points. **h** H3K27ac and H3K4me1 levels at enhancers in i-LADs, vLADs, and cLADs (all time points aggregated); bar, median; cross, mean; box, 25–75% percentile; whiskers, min-max. H3K27ac **P = 0.0049, ***P = 0.0002; H3K4me1 **P = 0.0023; unpaired two-tailed *t*-tests with Welch’s correction. **i** H3K27ac and H3K4me1 levels at enhancers of repressed cLAD genes (all time points aggregated)

Variable gene expression in LADs could also be attributed to genes being associated with enhancers located inside or outside LADs [7, 8]. To address this, we used the “double-elite” enhancer set of the GeneHancer database [29] to infer 2669 enhancers targeted to the DE genes found in all ten LAD classes (Fig. 8a). Among these, 908 harbor at least one H3K27ac peak at any time point in the 72-h time course and are thus most relevant in our adipogenic system (Fig. 8a, b; Additional file 2, Table S7).

We determined the LAD versus i-LAD localization of these enhancers as a function of time and of the LAD class of their target genes (Additional file 2, Table S7). We first find that enhancers targeting cLAD genes are in majority in LADs at all time points, and lose lamin association also when T00–T24 common LADs end up in i-LADs at 72 h (Fig. 8c, left 2 column sets). Second, the majority of enhancers of vLAD genes are located in i-LADs, whereas 30–40% follow the gained/lost LAD status of their target genes (Fig. 8c). This can be explained by the small size of vLADs (see Fig. 3c), which implies a strong likelihood of enhancers of vLAD genes to be outside LADs.

Co-segregation of enhancers and their target genes within LAD classes suggests linear proximity. In agreement with this, the mean distance between enhancers and the start site of their target genes is 24 kb (Fig. 8d, e), with 42% of these enhancers being in the gene body or downstream (Fig. 8f). In addition, H3K27ac and H3K4me1 signal strengths are stronger in enhancers localized in i-LADs compared to those in LADs (*P* = 2×10^−3^ to *P* = 2×10^−4^, unpaired two-tailed *t*-tests with Welch’s correction; Fig. 8g, h), suggesting stronger activity of i-LAD enhancers targeted to differentially expressed LAD genes. As expected, enhancers of repressed LAD genes do not harbor these marks (Fig. 8i). We infer from these results that association of enhancers with DE LAD genes is overall short-range. Further, overall co-partitioning of DE LAD genes with their enhancers in LADs or i-LADs is a feature of the early adipogenic gene expression program.

## Discussion

LADs are increasingly recognized genome organizers important for cell identity [3, 4]. We provide evidence of rearrangement of the genome in ASCs in the first 72 h of adipogenic differentiation, in the form of gain and loss of stand-alone LADs, and extension and shortening of edges of existing LADs. Strikingly, the fate of ASC LADs after adipogenic induction is pre-patterned by histone PTM signatures in low-LMNB1 sub-domains within LADs, including cLADs. The selective LAD versus i-LAD outcomes shown here reflect a reorganization of peripheral chromatin during early adipogenesis, restraining non-adipogenic genes in repressive LAD environments.

Cells from distant lineages share fewer cLADs than more closely related cell types, and cell type-specific LADs harbor ontologically distant cell type-specific gene expression signatures [4]. We show here that vLADs emerging sequester genes irrelevant for adipocyte functions, while genes involved in adipogenesis are released from lamin associations into a transcriptionally permissive environment. Even though we do not demonstrate a functional role of vLADs in the establishment of adipocyte identity, defective differentiation of ASCs expressing lipodystrophic lamin A/C mutations [30, 31] highlight the importance of a physiological nuclear lamin network in the commitment of ASCs to adipogenesis.

We cannot at present exclude that some LADs are repositioned stochastically shortly after induction of differentiation. In line with this view, vLADs are shorter than cLADs, they represent areas of lower lamin enrichment [16, 32] (this paper) and of more transient lamin interactions [33], and they do not strongly correlate with gene expression changes. Transient LADs and i-LADs may also reflect a large-scale destabilization of lamina-chromatin interactions facilitating a subsequent more determinative cell type-specific repositioning of LADs. They may also be a prerequisite for the (re)incorporation of non-adipogenic lineage-specific genes in LADs, and for the release of adipogenic genes from lamin constraints. Disruption of lamina interactions may precede a change in gene activity, “priming” a locus for activation [5, 6]. For example in our study, the *MAFB* gene, involved in the regulation of lineage-specific differentiation, is released from a LAD edge within 24 h without a change in expression, but is upregulated when cells engage into adipogenesis. Non-random repositioning of LADs by 72 h across domains that can be traced back in undifferentiated proliferating ASCs argues that LADs are intrinsic organizers of the genome in adipocyte progenitors. Our results support therefore a hypothesis of lineage commitment determined at least in part by LAD repositioning during adipogenesis.

Histone modifications around promoters of DE genes in LADs, such as H3K4me1/me3 and H3K27ac, also appear to predict LAD vs. i-LAD outcome during differentiation. Since these are already detected before differentiation onset, they seem to epigenetically shape the adipogenic gene expression program. This view is supported by DNA methylation [19] and chromatin accessibility states [24] at adipogenic promoters in undifferentiated ASCs, and by pre-established enhancer-enhancer connections specifying adipose versus osteogenic lineage determination in bone marrow mesenchymal stem cells [26]. Our findings imply that ASCs are epigenetically primed for adipogenesis and that chromatin states of DE genes in sub-domains of LADs with reduced LMNB1 occupancy anticipate LAD fate after adipogenic commitment.

A current view of maintenance of cell identity via LADs through cell division may provide insights into mechanisms of LAD positioning during differentiation. LADs have been proposed to be bookmarked by persistent H3K9me2 on mitotic chromosomes, which targets lamins during nuclear envelope reassembly [34]. Similarly, peripheral H3K9me2 or H3K9me3 domains in interphase nuclei could act as target sites for lamins. Supporting this view is the positioning of LADs on domains often pre-marked by H3K9me3 during adipogenesis (see Fig. 2c), and the persistence of H3K9me3 after a loss of LAD. Thus even though K3K9me3 enrichment is lower in vLADs than cLADs, domains of H3K9 methylation may underline a “LAD memory” of the adipose lineage.

At the nuclear periphery, H3K9me2 domains outside LADs have also been shown to constrain tissue-specific promoter-enhancer interactions [35] and could regulate gene expression in cLADs at sites of low lamin occupancy, or in vLADs released from the lamina. Locally reduced lamin-promoter contact frequencies may favor gene expression in LADs [14, 16, 32]. Interestingly, sites of chromatin accessibility in ASCs and adipocytes [24] coincide with low-LMNB1 H3K4me1/H3K27ac sites in cLADs (our unpublished data) and thus could facilitate enhancer-promoter interactions in LADs. Release of enhancers and genes from the nuclear lamina can ease their mutual interaction and promote cell type-specific activation [7, 8, 11]. Accordingly, enhancers of vLAD genes predominantly follow the LAD versus i-LAD fate of their target genes during ASC differentiation. Exploring space-time and functional relationships between LAD and i-LAD domains of the nuclear periphery may bring new insights on the regulation of cell type-specific gene expression during differentiation of adipocyte progenitors into various adipose lineages.

## Conclusions

LADs constitute predictable features of adipose cell nuclear architecture involved in sequestering non-adipogenic genes during adipocyte differentiation.

## Methods

### Adipose stem cell culture and adipose induction

Human ASCs were isolated from subcutaneous liposuction material as per protocol approved by the Norwegian Research Ethics Committee with No. 2013/2102. Cells were cultured in DMEM/F12 with 10% fetal calf serum and 20 ng/ml basic fibroblast growth factor. Proliferating ASCs were harvested and reseeded at confluency. Cells were cultured in a confluent state for 48 h in media without basic fibroblast growth factor and induced to differentiate at T00 with an adipogenic cocktail consisting of 10 μg/ml insulin, 200 μM indomethacin, 1 μM dexamethasone, and 0.5 μM 3-isobutyl-1-methylxanthine. Cells were harvested for analyses before differentiation (T00) and at T04, T08, T12, T16, T20, T24, and T72.

### Cell proliferation assay

ASCs were stained for proliferation assay in two independent experiments with CellTrace Violet using the CellTrace™ Violet Cell CFSE Proliferation Kit (C34571, ThermoFisher). Proliferating ASCs were harvested and stained with carboxyfluorescein succinimidyl ester (CFSE). A batch was analyzed by flow cytometry (NovoSample Pro, Agilent) while the rest was plated confluent at 25,000 cells/cm^2^ in media without basic fibroblast growth factor 48 h before induction of differentiation (day 0). On day 0 (T00), 1 (T24), 2 (T48), and 3 (T72) cells were analyzed by flow cytometry along with control unstained cells. Cell counts were plotted as a function of CFSE intensity and data expressed as percent of dividing cells (M2 values on plots); data were analyzed using the NovoExpress package from Agilent (https://www.agilent.com/en/product/research-flow-cytometry/flow-cytometry-software/novocyte-novoexpress-software-1320805).

### Immunoblotting

Proteins were separated by 4–20% SDS-PAGE (H3K27ac, H3K4me3, H3K4me1, H3, γ-tubulin) or 10% SDS-PAGE (LMNB1, γ-tubulin) and transferred to an Immobilon-FL membrane (Millipore). Membranes were blocked with Odyssey blocking buffer (LI-COR) (H3K27ac, H3K4me3, H3K4me1, H3) or 5 % milk (LMNB1, γ-tubulin) and incubated with antibodies against H3K4me1 (1:500; Abcam ab8895), H3K27ac (1:1000; Abcam 177178), H3K4me3 (1:1000; Diagenode Mab-152-050), H3 (1:1000; Abcam ab1791), LMNB1 (1:1000; Santa Cruz Biotechnology sc6216), or γ-tubulin (1:10000; Sigma-Aldrich T5326). Proteins were detected with IRDYE-800-coupled antibodies or Peroxidase-conjugated antibodies. Relative protein levels were quantified using Image Lab (BioRad) (https://www.bio-rad.com). Uncropped Western blots are shown in Additional file 1, Fig. S8.

### RNA-sequencing

Total RNA was isolated from duplicate differentiation experiments using the RNAeasy mini kit (Qiagen) and processed for Illumina library preparation. RNA-seq reads were aligned to hg38 and duplicates removed using Picard MarkDuplicates (http://broadinstitute.github.io/picard/). Transcript abundance was estimated using --featureCounts in Subread v2.0.1 (http://subread.sourceforge.net) [36]. We defined an expressed gene as a gene with a trasncript normalized read count of ≥ 15. Differential gene expression (DE; FDR ≤ 0.05) was determined using Bioconductor DESeq2 v1.30.0 (https://bioconductor.org/packages/release/bioc/html/DESeq2.html) with default parameters as implemented in SARTools (https://rdrr.io/github/PF2-pasteur-fr/SARTools/man/SARTools-package.html) [37]. Reads per kilobase of transcript per million mapped reads (RPKM) were calculated for each transcript. Protein-coding genes identified as differentially expressed between at least two consecutive time points were used for cluster analysis. A Dirichlet process Gaussian process mixture model clustering (https://github.com/PrincetonUniversity/DP_GP_cluster) [38] was applied to define expression clusters. Heatmaps were generated by clustering expression *z-*scores using Ward’s method in the R function pheatmap (https://biocorecrg.github.io/CRG_RIntroduction/heatmap-2-function-from-gplots-package.html). GSEA was done on normalized read counts [39]. Gene ranking was generated across all time points with Pearson correlation metric and analyzed against the mSigDB Hallmarks v7.5.1 gene sets [40].

### Chromatin immunoprecipitation (ChIP)

ChIP of H3K27ac, H3K4me3, and H3K4me1 was done as described in [6]. In brief, cells were fixed with 1 % formaldehyde; lysed in 50 mM Tris-HCl, pH 8, 10 mM EDTA, 1% SDS, protease inhibitors, and Na-butyrate; and sonicated in a Biorupter (Diagenode) into ~200 base-pair fragments. After sedimentation, the supernatant was diluted 10 times and chromatin incubated with anti-H3K27ac (Diagenode c15410174), anti-H3K4me3 (Diagenode c15410003) or anti-H3K4me1 (Diagenode c15410037) antibodies, each at 2.5 μg/10^6^ cells, for 2 h at 4°C. ChIP samples were washed, cross-links reversed, and DNA eluted for 2 h at 68°C. DNA was purified using phenol-chloroform isoamylalcohol and dissolved in H_2_O. Libraries were prepared using a Microplex kit (Diagenode) and sequenced on a Nextseq 500 or Novaseq (Illumina).

### ChIP-sequencing analysis of histone modifications

FASTQ sequences from H3K27ac, H3K4me3, and H3K4me1 ChIPs were aligned to hg38 using Bowtie2 v2.4.1 [41]. Duplicate reads were removed as above and peaks detected using MACS2 v2.2.7.1 (https://github.com/macs3-project/MACS/releases/tag/v2.2.7.1) [42]. For H3K4me1, peaks were called from both replicates. Log2(ChIP/Input) ratios were calculated using bamCompare in Deeptools v3.5.1 (https://github.com/deeptools/deepTools/releases/tag/3.5.1) [43]. ChIP read counts were normalized to library size using the --reads per genome coverage function in Deeptools. Bigwig files for normalized read counts were visualized using Integrative Genomics Viewer (https://software.broadinstitute.org/software/igv/) [44]. H3K9me3 ChIP-seq datasets genereated by us [22] were downloaded from NCBI Gene Expression Omnibus (GEO) GSE109924 [45]. Reads were aligned to hg38 and bam files down-sampled between ChIP and Input. Bigwig tracks were generated from Log2(Chip/Input) ratios in 1-kb bins using bamCompare from Deeptools. ggplot2 in R (https://ggplot2.tidyverse.org/) was used for plots.

### Mapping LADs from LMNB1 ChIP-seq data

LMNB1 ChIP-seq and input reads were previously generated by us [22] from two adipogenic differentiations using the same batch of ASCs as in this study and downloaded from NCBI GEO GSE109924 [45]. Reads were aligned to hg38 after removing duplicates. To avoid normalization bias, each pair of mapped ChIP and input read files contained the same read depth by down-samplings read for each chromosome. To assess ChIP data quality and reproducibility, Pearson correlations were determined between replicates from Log2(LMNB1/Input) ratios in 100-kb bins across the genome using Deeptools. Mapped reads were used to call LADs using ten runs of Enriched Domain Detector (http://github.com/CollasLab/edd) [46] with auto-estimation of GapPenalty and BinSize, and mean GapPenalty and BinSize values from these runs were used for a last run. Final LADs were the union of LADs of both replicates at each time point. Pearson correlations between replicates were also calculated for LMNB1 ChIP read counts within the merged LADs.

### Gene ontology enrichment analysis

GO enrichment (GO biological process) was analyzed using Protein ANalysis THrough Evolutionary Relationships (PANTHER) v.14.0 (http://www.pantherdb.org/) [47].

### Intersections between LADs, genes, histone PTMs, and enhancer positions

Intersects between LADs, genes, and histone PTMs were determined using BEDTools v2.29.2 (https://github.com/arq5x/bedtools2/releases/tag/v2.29.2) [48] and BEDOPS v2.4.37 (https://github.com/bedops/bedops/releases/tag/v2.4.37) [49]. A gene was ascribed to a LAD if it overlapped with the LAD by at least one base-pair. Genes spanning more than one LAD class were removed from the analyses. Intersects between LADs and H3K27ac enhancers were determined using Intervene v0.6.4 (https://github.com/asntech/intervene/releases/tag/0.6.4) [50]. Enhancers were the “double-elite” set of the GeneHancer database, containing high-likelihood enhancers with strong enhancer-gene associations (https://www.genecards.org) [29].

Mean Log2(LMNB1/Input) from all time points confounded was calculated using wiggletools v1.2 (https://github.com/Ensembl/WiggleTools) [51]. H3K4me1 peaks were merged from all time points and intersected with cLADs using BEDOPS. Mean Log2(LMNB1/Input) of the H3K4me1 intersected and non-intersected region was calculated using multiBigwigSummary from Deeptools.

### Statistical tests of LAD genomic overlaps

To test the overlap of T00-T24 LAD categories with LADs at T72 (Fig. 4c), and the overlap of the ten LAD classes with LADs in proliferating ASCs (Fig. 4e), we used the Overlap function of the Genomic HyperBrowser [52] (https://hyperbrowser.uio.no); this function tests the null-hypothesis that domains are independently positioned relative to each other in a target genome. Each LAD from a given chromosome was permuted 50 times across the same chromosome in cells at T72, or 250 times in proliferating ASCs using as background the union of all known LAD locations in these cells. *P*-values were from two-sided permutation tests with FDR ≤ 0.1. Permutation runs are available at https://hyperbrowser.uio.no/hb/u/mohamedabdelhalim/h/d0d1ladsinterd3) and at (https://hyperbrowser.uio.no/hb/u/mohamedabdelhalim/h/dm2inter10ladclass). For comparisons of overlap between LAD classes (Fig. 4f), *P*-values were from Fisher’s tests with two-tailed *P*-values.

## Supplementary Information

**Additional file 1: Table S2.** LAD class description over the 72-h differentiation time course. **Table S3.** Observed and expected overlaps of T00-T24 LAD categories with LADs at 72 h. **Table S4.** Observed and expected overlap of each LAD class with LADs in undifferentiated proliferating ASCs. **Table S6.** Summary of GO terms in LAD classes. **Figure S1.** Cell proliferation and LMMB1 levels during early adipogenic differentiation. **Figure S2.** RNA-seq transcription profiling of early adipogenesis. **Figure S3.** Profiles of genomic LMNB1 enrichment during differentiation. **Figure S4.** Characteristics of the LAD classes. **Figure S5.** Expression profiles of individual DE genes in LAD classes. **Figure S6.** H3K4me3, H3K27ac and H3K4me1 levels during differentiation. **Figure S7.** H3K4me3, H3K27ac and H3K4me1 levels and patterns around TSSs of genes localized in lost or gained edges during differentiation. **Figure S8.** Uncropped Western blots.

**Additional file 2: Table S1.** Differentially expressed genes in each expression cluster. **Table S5.** Protein-coding genes uniquely found in each LAD class. **Table S7.** Location of enhancers targeted to DE genes in each LAD class.

**Additional file 3.** Review history.

## Data Availability

RNA-seq data and H3K4me1, H3K4me3, and H3K27ac ChIP-seq data generated and used in this study are available at NCBI GEO with accession number GSE185066 (https://www.ncbi.nlm.nih.gov/geo/query/acc.cgi?acc=GSE185066) [53]. Our third-party LMNB1 and H3K9me3 ChIP-seq datasets re-analyzed in this study are available at NCBI GEO with accession number GSE109924 (https://www.ncbi.nlm.nih.gov/geo/query/acc.cgi?acc=GSE109924) [45]. Enhancers used in this study were the double-elite set from GeneHancer (https://www.genecards.org) [29].
