# Supplementary material for: Local euchromatin enrichment in lamina-associated domains anticipates their repositioning in the adipogenic lineage
Source: Genome Biol. 2022 Apr 11;23:91. doi: 10.1186/s13059-022-02662-6 (PMC8996409; doi:10.1186/s13059-022-02662-6)
Supplement: Supplementary file 1 — Additional file 1: Table S2. LAD class description over the 72-h differentiation time course. Table S3. Observed and expected overlaps of T00-T24 LAD categories with LADs at 72 h. Table S4. Observed and expected overlap of each LAD class with LADs in undifferentiated proliferating ASCs. Table S6. Summary of GO terms in LAD classes. Figure S1. Cell proliferation and LMMB1 levels during early adipogenic differentiation. Figure S2. RNA-seq transcription profiling of early adipogenesis. Figure S3. Profiles of genomic LMNB1 enrichment during differentiation. Figure S4. Characteristics of the LAD classes. Figure S5. Expression profiles of individual DE genes in LAD classes. Figure S6. H3K4me3, H3K27ac and H3K4me1 levels during differentiation. Figure S7. H3K4me3, H3K27ac and H3K4me1 levels and patterns around TSSs of genes localized in lost or gained edges during differentiation. Figure S8. Uncropped Western blots. [file 13059_2022_2662_MOESM1_ESM.pdf]

Additional file 1

Table S1. Differentially expressed genes in each expression cluster. See **Additional file 2** (Excel).

Table S2. LAD class description over the 72 h differentiation time course.

T00-T24	T72	No. domains	Coverage (Mb)	No. genes*	Genes* per Mb	% TSSs in LAD	No. expressed genes*§	No. DE genes*‖
Lost LAD	LAD	21	9.7	30	3.1	100	8	7
	i-LAD	32	9.0	49	5.4	96	20	17
Gained LAD	LAD	98	52.6	177	3.3	98	25	14
	i-LAD	150	50.9	204	4.0	93	54	44
cLAD	LAD	379	505.7	653	1.3	98	72	72
	i-LAD	326	52.2	174	3.3	99	44	36
Lost edge	LAD	173	15.0	30	2.0	90	17	6
	i-LAD	254	28.4	191	6.7	81	90	81
Gained edge	LAD	245	60.3	80	1.3	1000	18	12
	i-LAD	295	49.3	246	5.0	91	106	105

*Protein-coding genes unique to each LAD class.
§Expressed genes are genes expressed at ≥ 1 time point in the 0-72 h time course.
‖DE, differentially expressed (FDR ≤ 0.05).

Table S3. Observed and expected overlaps of T00-T24 LAD categories with LADs at 72 h.

LAD category	Total cov. (Mb)	Observed overlap (Mb)	% Observed	Expected overlap (Mb)	% Expected	FDR-adj. P-value
Lost LAD	18.8	9.72	51.7	3.81	20.3	0.01961
Gained LAD	103.5	52.61	50.8	24.11	23.3	0.01961
Common LAD	557.8	505.77	90.7	140.60	25.2	0.01961
Lost edge	43.4	15.02	34.6	10.69	24.6	0.01961
Gained edge	109.7	60.33	55.0	27.63	25.2	0.01961

Each LAD was permuted 50 times across the same chromosome using as background the union of all known LAD locations on the same chromosome in cells at T72; P-values are from two-sided permutation tests with FDR correction (≤ 0.1) for multiple testing. Numbers of permutations are set by the 'Overlap' function of the Genomic HyperBrowser (https://hyperbrowser.uio.no) used to perform permutations.

Table S4. Observed and expected overlap of each LAD class with LADs in undifferentiated proliferating ASCs.

LAD category	Total cov. (Mb)	Observed overlap (Mb)	% Observed	Expected overlap (Mb)	% Expected	FDR-adj. P-value
Lost LAD > LAD	9.73	2.01	20.6	2.36	24.28	0.6255
Lost LAD > i-LAD	9.03	0.25	2.7	1.44	15.95	0.9721
Gained LAD > LAD	52.61	28.11	53.4	13.44	25.55	0.003984
Gained LAD > i-LAD	50.92	20.51	40.3	11.03	21.66	0.003984
Common LAD > LAD	505.78	436.89	86.4	127.5	25.21	0.003984
Common LAD > i-LAD	52.02	28.96	55.7	13.03	25.05	0.003984
Lost edge > LAD	15.03	7.21	47.9	3.81	25.32	0.003984
Lost edge > i-LAD	28.42	8.88	31.3	7.08	24.90	0.08367
Gained edge > LAD	60.33	49.28	81.7	16.26	26.95	0.003984
Gained edge > i-LAD	49.37	30.98	62.7	12.48	25.28	0.003984

Each LAD was permuted 250 times across the same chromosome using as background the union of all known LAD locations on the same chromosome in proliferating ASCs; P-values are from two-sided permutation tests with FDR correction (≤ 0.1) for multiple testing. Numbers of permutations are set by the 'Overlap' function of the Genomic HyperBrowser (https://hyperbrowser.uio.no) used to perform permutations.

Table S5. Protein-coding genes uniquely found in each LAD class. See **Additional file 2** (Excel).

Table S6. Summary of GO terms in LAD classes.

LAD class	GO term (PANTHER, biol. Process)	P-value*
Lost LAD > LAD (i)	Neuronal differentiation	5.7E-05
	Kidney cell function	4.3E-03
	Cardiac function	4.6E-03
	Pancreas differentiation	8.7E-03
	Liver development	1.0E-02
Lost LAD > iLAD (ii)	Steroid metabolism	3.6E-04
	Transcription	1.3E-04
	Fatty acid synthesis	1.3E-02
Gained LAD > LAD (iii)	Detection of stimulus	5.5E-19*
	GPRC signaling	1.5E-11*
	Cellular metabolic process	6.2E-10*
Gained LAD > iLAD (iv)	Detection of stimulus	1.5E-28*
	GPCR signaling	1.1E-15*
	Cellular metabolism	4.6E-04*
	Regulation of transcription	1.5E-03*
cLAD > LAD (v)	Nervous system process	1.4E-13*
	GPCR signaling	1.7E-13*
	Cellular metabolic process	4.3E-12*
	System process	1.7E-11*
cLAD > iLAD (vi)	Detection of stimulus	4.5E-29*
	Nervous system process	8.2E-17*
	GPCR signaling	1.0E-15*
Lost edge > LAD (vii)	Regulation of cardiac function	3.4E-04
	Canonical Wnt signaling	2.9E-03
	Developmental cell growth	7.7E-03
	Mesodermal cell migration	8.7E-03
Lost edge > iLAD (viii)	Immune system process	3.6E-04
	Organelle inheritance	5.8E-03
	Fat cell differentiation	1.6E-02
	Lipid phosphorylation	9.9E-03
Gained edge > LAD (ix)	Metabolic process	2.1E-04
	Mitochondrial function	2.2E-04
	Brown fat cell differentiation	7.2E-03
Gained edge > iLAD (x)	GPCR signaling	8.8E-04
	Oxidative phosphorylation	3.3E-03
	Cellular metabolic process	7.6E-03

*Fisher's exact tests with FDR ≤ 0.05; no star: no FDR correction. GO terms are for all genes in each LAD class irrespective of their expression status.

Table S7. Location of enhancers targeted to DE genes in each LAD class. See **Additional file 2** (Excel).

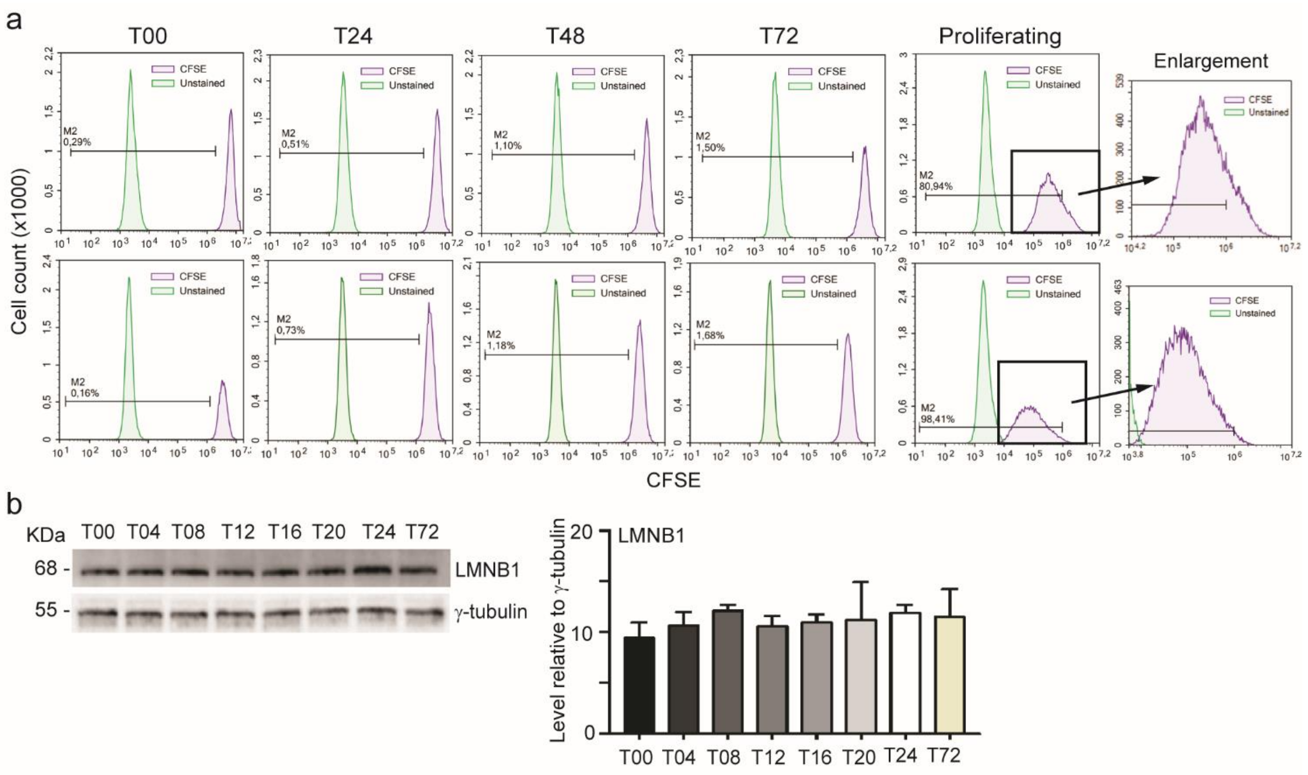

Figure S1. Cell proliferation and LMMB1 levels during early adipogenic differentiation. **a** Cell proliferation monitored by carboxyfluorescein succinimidyl ester (CFSE) during differentiation. Plots show cell counts as a function of CFSE fluorescence intensity in unstained and stained cells at indicated time points; data for two differentiation replicates (upper and lower rows). Proliferating ASCs were used as a dividing cell population control. M2, percentage of dividing cells in the population. **b** Western blot analysis of LMNB1 protein expression during differentiation; γ-tubulin was used as loading control; left, Quantification of LMNB1 Western blots as in (a) relative to γ-tubulin (n = 2 differentiations).

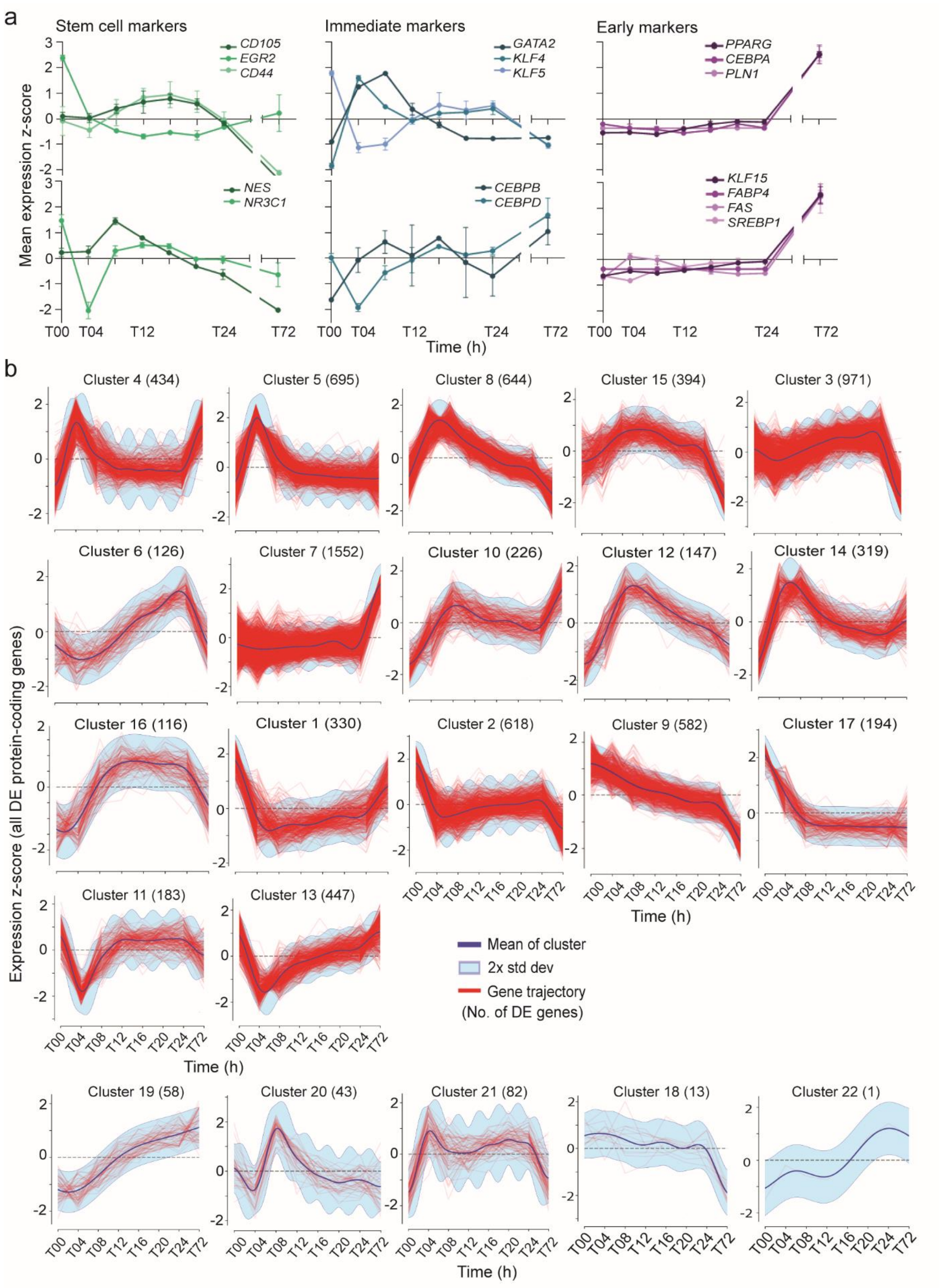

Figure S2. RNA-seq transcription profiling of early adipogenesis. **a** Expression z-score of stem cell, immediate and early adipogenic genes in the first 24 h of differentiation, and at 72 h; mean ± SD of two differentiations. **b** Unsupervised gene expression clusters of all DE genes (No. of genes in cluster); clusters are ordered by row based on profiles.

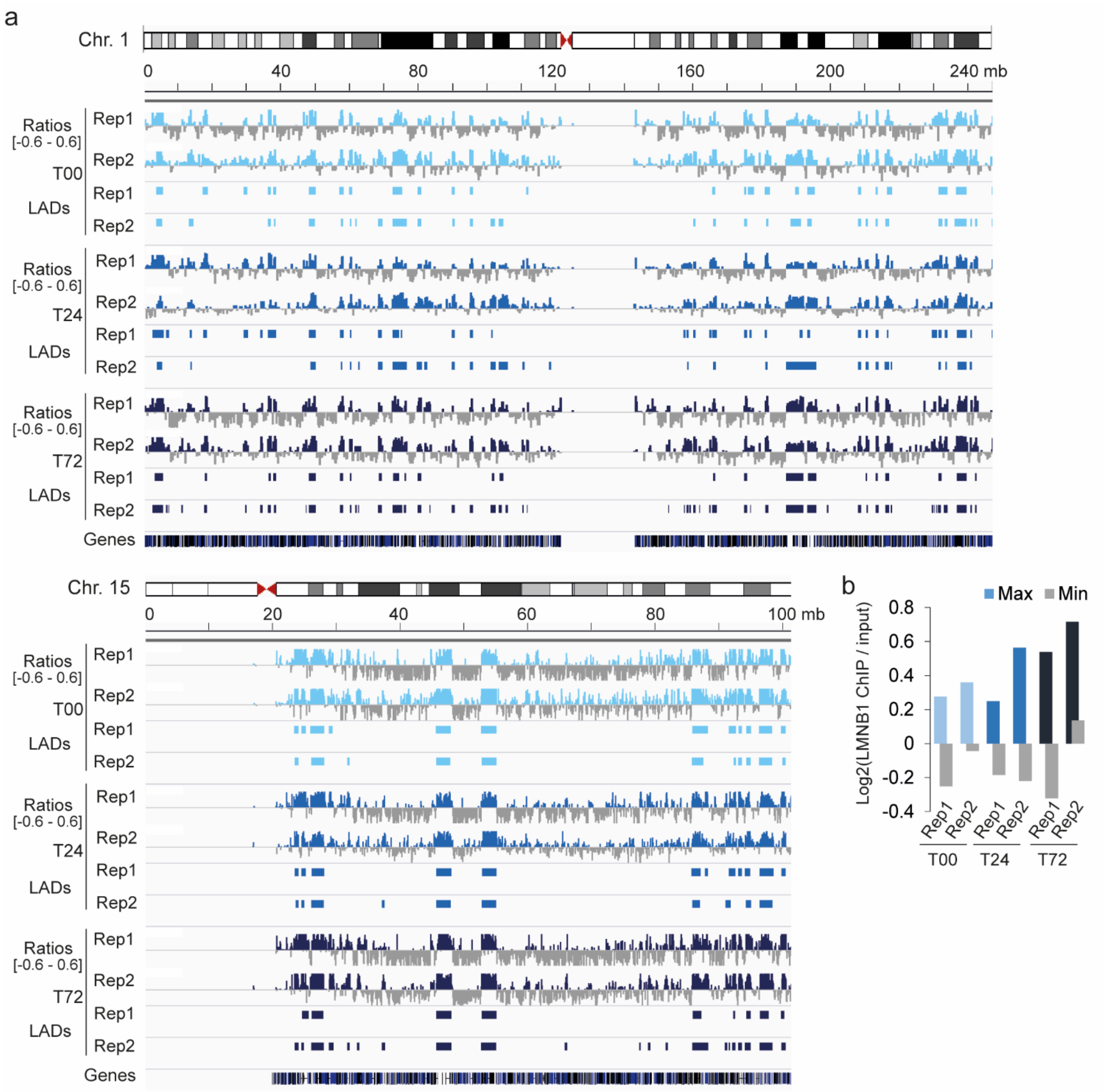

Figure S3. Profiles of genomic LMNB1 enrichment during differentiation. **a** Genome browser views of LMNB1 ChIP enrichment as Log2(ChIP/input) ratios and called LADs across chromosomes 1 and 15 for each differentiation replicate (Rep1, Rep2) at T00, T24 and T72; Log2(ChIP/input) ratio scale is shown in brackets. **b** Dynamic range of LMNB1 ChIP/input ratios (Log2) in each differentiation replicate.

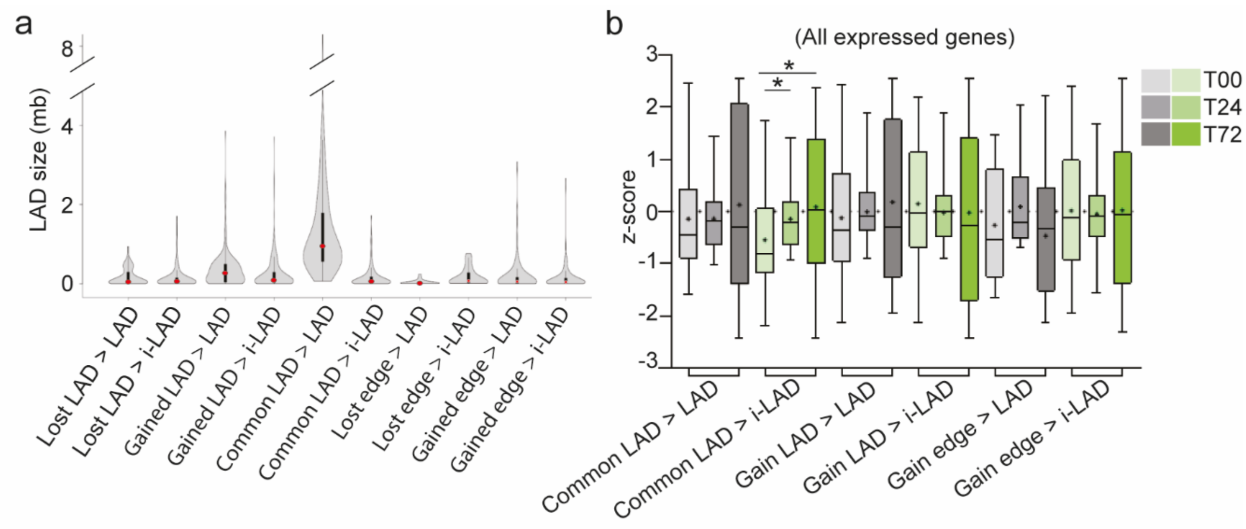

Figure S4. Characteristics of the LAD classes. **a** Violin and box plots of LAD size distribution in each LAD class; box plot: dot, median; thick bar, 25-75% percentile; whiskers, min-max. **b** Expression z-score of all expressed genes in indicated LAD classes at T00, T24 and T72. Bar, median; cross, mean; box, 25-75 percentile, whiskers min-max; *P = 0.02, unpaired two-tailed t-test with Welch's correction.

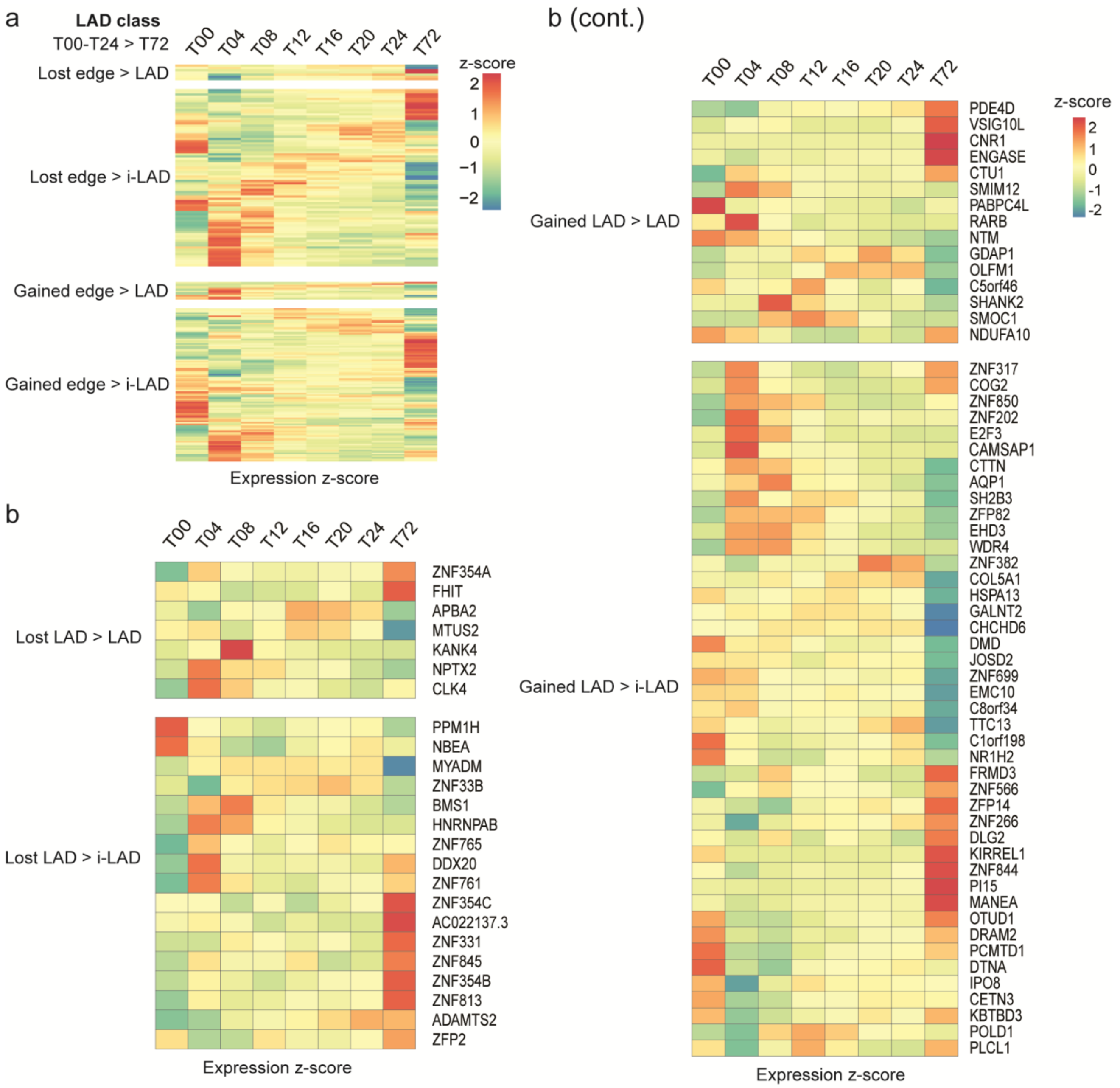

Figure S5. Expression profiles of individual DE genes in LAD classes. **a** Hierarchically clustered heatmaps of expression z-scores, scaled across time points in Lost/Gained edge LAD classes. **b-c** Clustered heatmaps of expression z-scores detailed for each gene, scaled across time points. Figure continues on the next two pages.

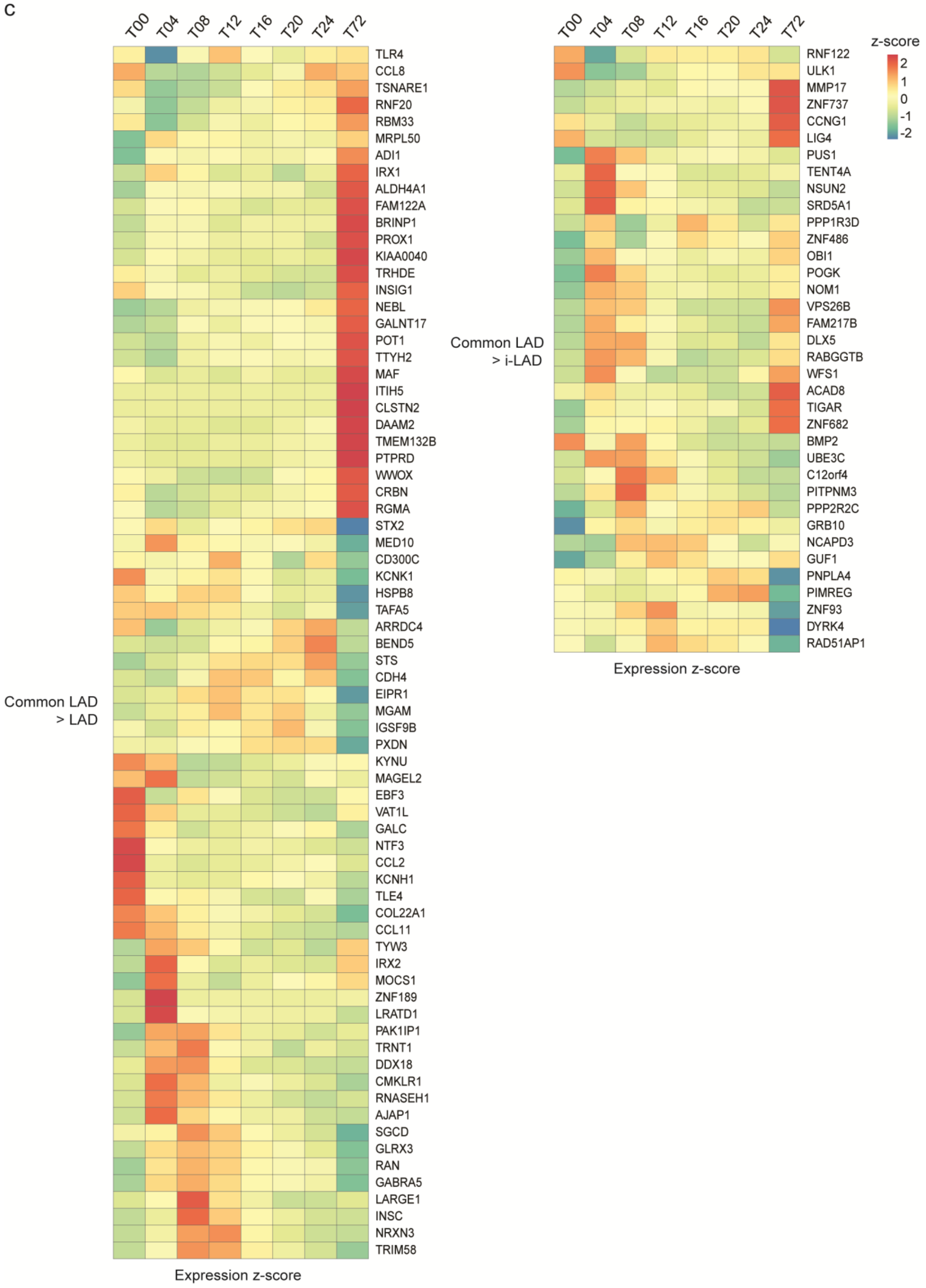

Figure S5 (cont.) Expression profiles of individual DE genes in each LAD class.

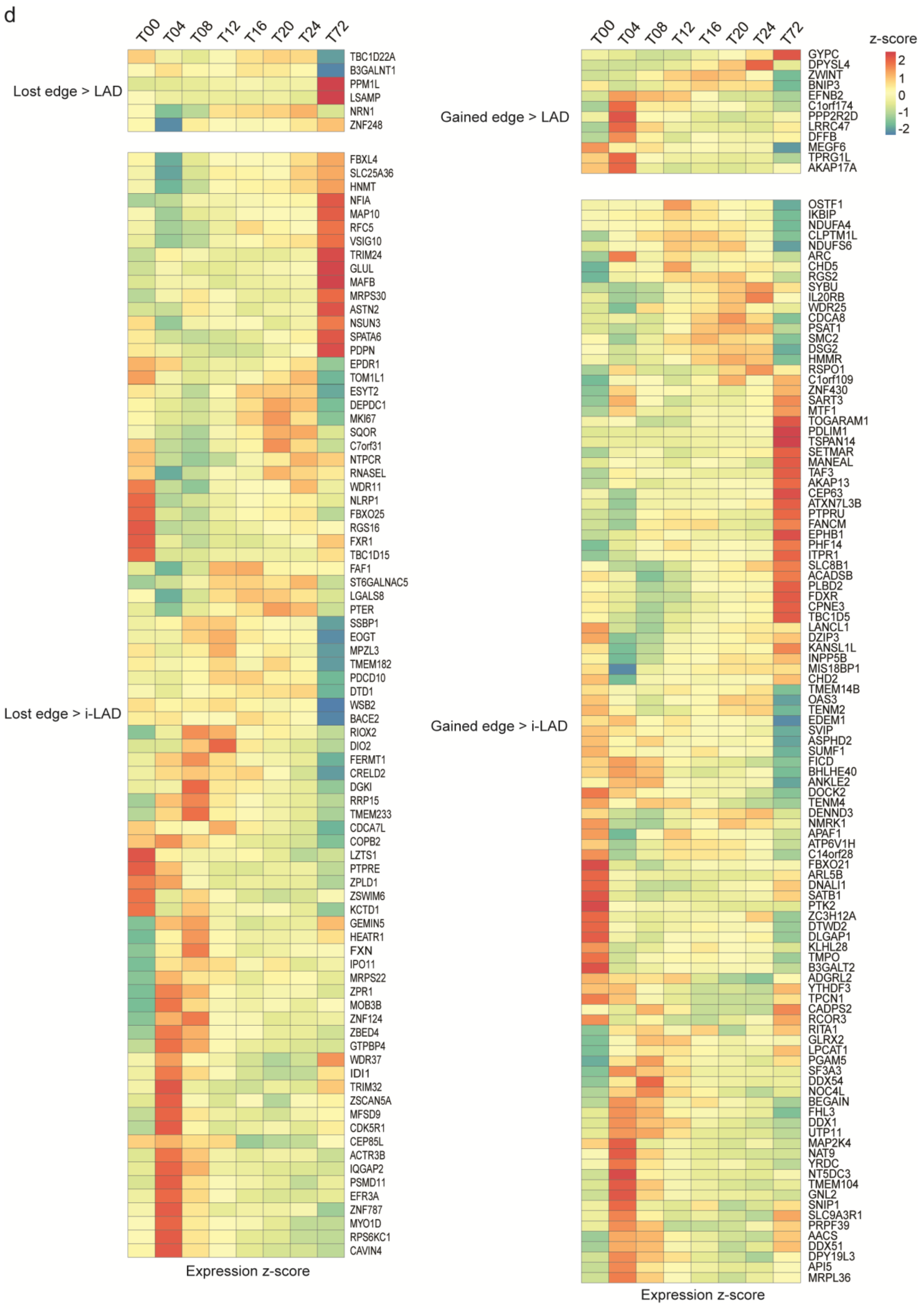

Figure S5 (cont.) Expression profiles of individual DE genes in each LAD class.

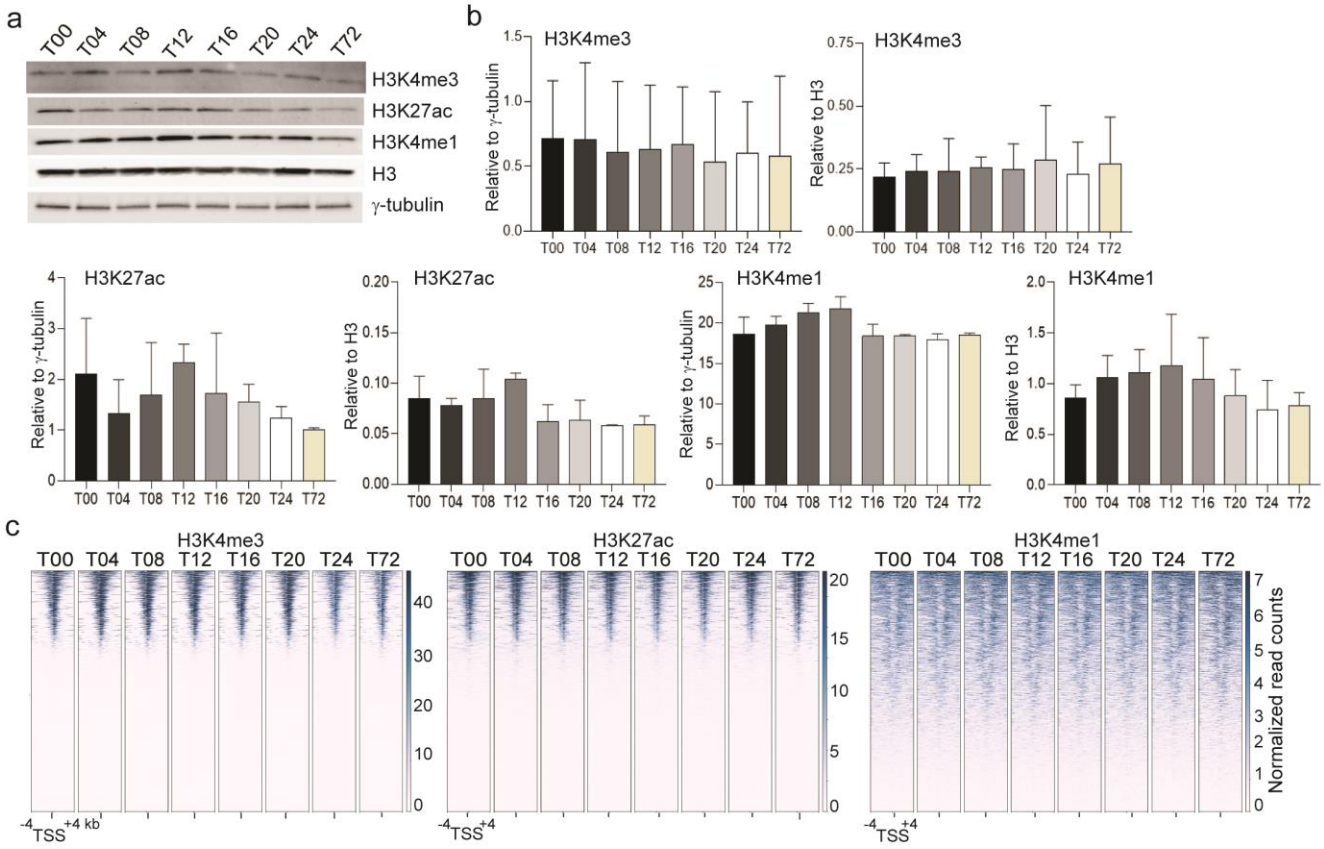

Figure S6. H3K4me3, H3K27ac and H3K4me1 levels during differentiation. **a** Western blot of total H3K4me3, H3K27ac and H3K4me1 at each differentiation time point; histone H3 and γ-tubulin were used as loading controls. **b** Quantification of Western blots as in **a** relative γ-tubulin and total H3; mean ± SD (min-max) from 2 differentiations; no significant differences are detected between time points. **c** Heatmaps of H3K4me3, H3K27ac and H3K4me1 distribution around the TSS ± 4 kb in the 0-72 h differentiation time course; read counts normalized to library size.

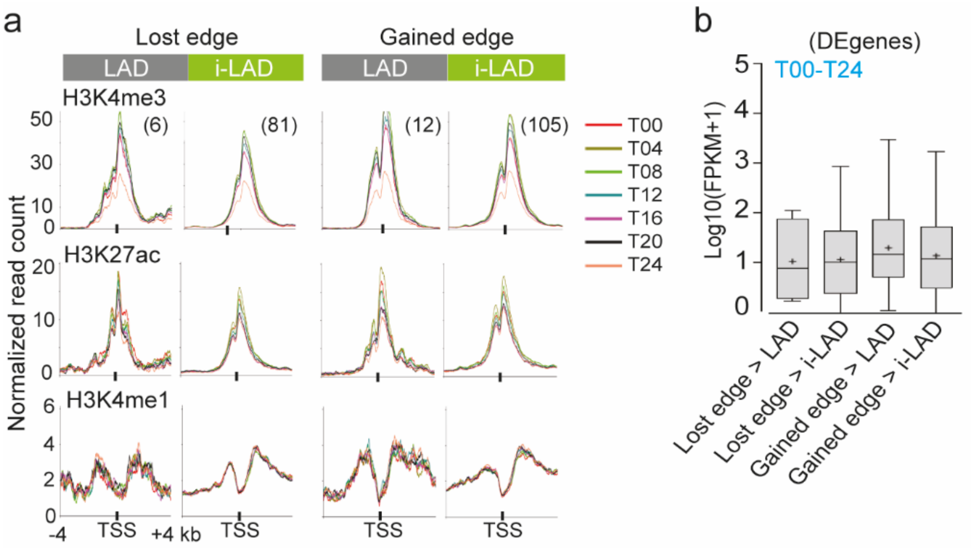

Figure S7. H3K4me3, H3K27ac and H3K4me1 levels and patterns around TSSs of genes localized in lost or gained edges during differentiation. **a** H3K4me3, H3K27ac and H3K4me1 levels at the TSS ± 4 kb in the T00-T24 time course; data are shown as mean ChIP-seq read counts normalized to library size. **b** Expression level of DE genes in lost and gained edge classes, averaged across the T00-T24 time course; bar, median; cross, mean; box, 25-75% percentile; whiskers, min-max. There are no significant differences between these LAD classes.

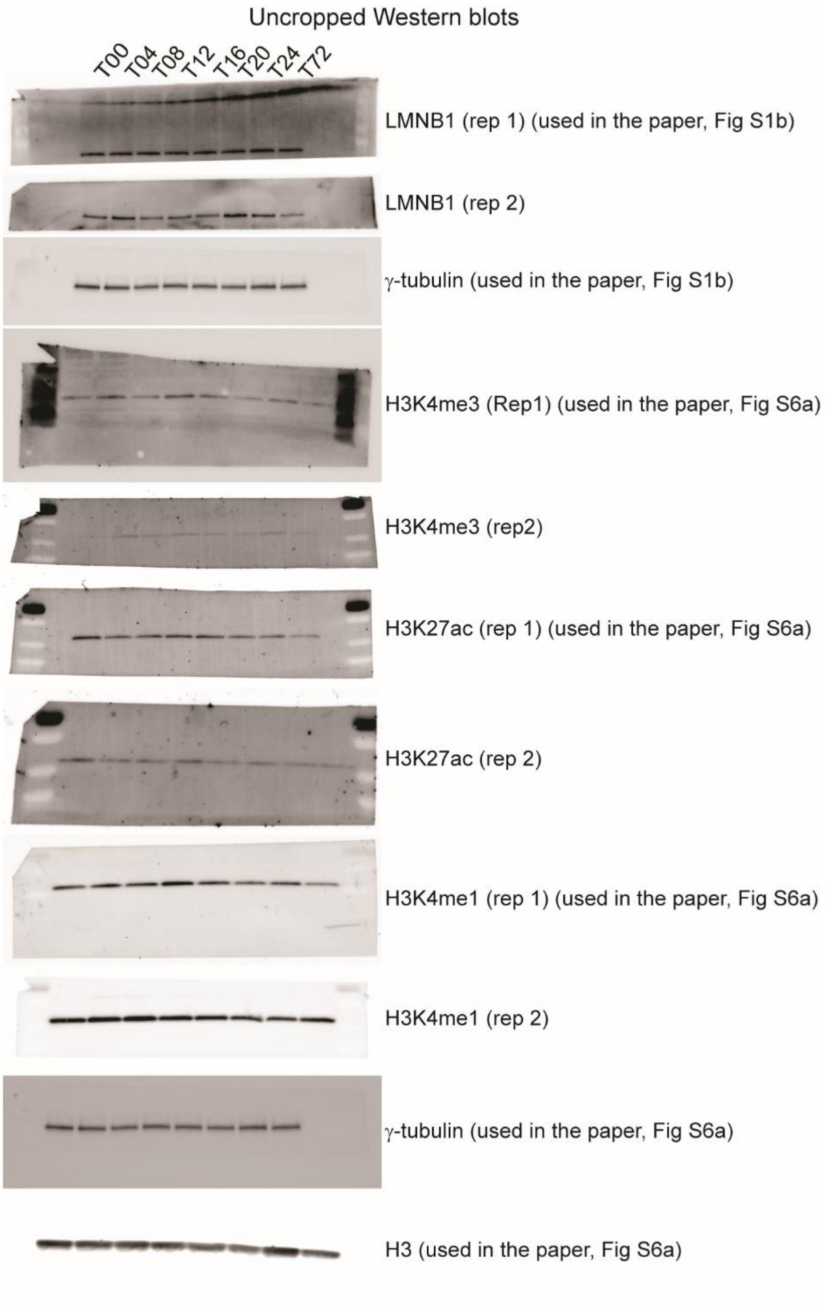

Figure S8. Uncropped Western blots.
