# Supplementary material for: Local euchromatin enrichment in lamina-associated domains anticipates their repositioning in the adipogenic lineage
Source: Genome Biol. 2022 Apr 11;23:91. doi: 10.1186/s13059-022-02662-6 (PMC8996409; doi:10.1186/s13059-022-02662-6)
Supplement: Supplementary file 3 — Additional file 3. Review history. [file 13059_2022_2662_MOESM3_ESM.docx]

**1^st^ round**

**Reviewer 1**

Summary:

Madsen-Østerbye describe the LADs dynamic during adipose differentiation and correlate that with changes in transcription and enhancer-chromatin features. They use ChIP-seq data for LB1 from a previous paper (Paulsen Nat Gen2019) from the same group generated in ASC induced for adipose differentiation at 24 and 72 hours. They also generate new LB1 Chip-seq tracks for proliferating ASC, and maps for H3K4me1/3 and H3K27ac during differentiation. The ASC system provides a nice setting to study direct repositioning of LADs during differentiation without confounding effects like repositioning after mitosis.

They identified 10 different categories of LADs according to the kind of change (border vs full LADs), the direction of modulation at T24 (gain vs loss at T24) and the final location of the loci at T72 (LAD vs iLAD at T72). These variable LADs categories tend to overlap in ASC proliferating progenitors suggesting a sort of "LAD memory". Gene ontology analysis revealed that genes that end in LADs at T72 tend to be non adipogenic genes and genes in iLAD (72) resemble more an adipogenic program. This is independent of the LAD/iLAD trajectories at T24.

RNAseq indicates that changes in gene expression do not correlate with LAD dynamics.

ChIP-seq data for H3K4me3, H3K4me1 and H3K27ac showed that the presence of this mark could be predictive of the fate of the gene in LAD/iLAD at T72. DE genes that end in iLAD show higher level than LAD fate genes for these active marks (H3K4me3 and H3K27ac) even at T00, suggesting a priming/epigenetic effect on LAD fate. For H3K4me1 they see stronger depletion for these mark in iLAD fate DE genes at TSS.

In LADs, regions of H3K4me1 show reduced lamina interactions.

Tracking of enhancers positioning along differentiation revealed that enhancers imputed to cLAD genes are mainly located in cLADs, suggesting proximity to their cognate genes. Furthermore, most part of the enhancers associated to genes in vLADs reside outside LADs at any timepoint, while a minor proportion follows the LAD/i-LAD fate of their cognate genes.

Major points:

1) We (BvS and a lab member) independently found the manuscript very dry and difficult to read. It provides some interesting points regarding the enhancers or the LAD dynamics, with the caveat that the enhancer-gene links are imputed. But overall, the reported findings seem incremental, strictly descriptive, and of limited novelty. The datasets may be useful for the community, but the most interesting ChIP-seq maps of lamina interactions were already published previously.

2) Often the text is confusing and we had to read several paragraphs multiple times to understand them. It was often difficult to follow the logic of the experiments/analyses and the underlying rationale.

3) One reason why the results are difficult to digest is the choice of the authors to categorize LAD changes in 10 different categories spanning three different time points. Although we recognize the need to properly call LAD changes (border vs LAD, gain vs extension, loss vs shortening) one could question if this division is really necessary throughout the manuscript, especially considering that gene expression and enhancer position do not strictly depend on any of these categories. Could some of the main findings of the manuscript not be described by simply calculating an LB1 score per gene or enhancer?

4) We have some concerns about the quality of LB1-ChIP seq data and the application of the EDD algorithm. Please show original ChIP-seq tracks of LB1 in Figure 1a, instead of binary LAD tracks only. Please also provide an estimate of the quality of the ChIP tracks (signal to noise ratio, dynamic range, correlation between replicates). This is important because LAD borders and variable LADs are usually marked by low level of LB1 and its definition by an algorithm is subjected to fluctuation because of noise. The only way to minimize this is to increase the number of replicates, while in this study only two reps are present. Some of these replicates yield very different numbers, like T72 rep1 vs rep2 (Table S1) where the number of identified LADs and the size of LADs is extremely different between the two replicates.

This is even more complicated by the fact that the authors perform the EDD calling on the union of the two replicates. This suggest that gain and losses (for border or stand-alone LADs) can basically derive from one single replicate. More replicates should be added or at least intersection analysis should be performed. Also, the authors show only one track for LB1 chip and it does not seem of the highest quality.

Minor points:

1) The discussion could be further developed by a deeper interpretation of the data. For example: The dynamics at t24 (transient lamina association) are interesting (transient LADs vs transient iLAD) and could be discussed further. What would be the function of this transient repositioning to the lamina if it cannot be connected to gene expression and only marginally to enhancers?

2) In line 8 page 8 the authors say "Resilience of DE genes to changes in LAD status and to the overall heterochromatic environment of cLADs. To claim that the authors should provide a quantitative analysis of H3K9me3 during differentiation

3) In legend of figure 5 the letter "a" is repeated twice and "RNA-seq profiling of early adipogenic differentiation" is either misplaced or absent from the figure.

4) Boxplot and violinplot in fig1C are not aligned.

==========Reviewed by Bas van Steensel and a member of the BvS lab. Review assignment accepted 15 Oct 2021, completed 25 Oct 2021. It is BvS's standard policy to disclose his identity in *all* of his manuscript reviews, irrespective of his comments and recommendations. All correspondence should be addressed to the Editor. PLEASE DO NOT REMOVE THIS NOTE. ==========

**Reviewer 2**

The dynamics of chromatin association with the nuclear lamina during development is incompletely understood. The relationship between LAD dynamics and gene expression during differentiation and development remains elusive. In this manuscript, the authors defined the dynamics of Lamin B1-genome interactions during adipogenic differentiation and compared it with the dynamics of gene expression and histone modifications. The authors reported an extensive modulation of LADs during the adipogenic stem cell differentiation. The authors uncovered various genomic features such as LAD association, TSS histone mark states, local lamina-association levels, locations of the enhancers with respect to LADs, that correlate with expression dynamics of genes within LADs during the differentiation. One important and interesting finding from these analyses is that adipogenic gene expression changes are correlated with genomic features other than lamina association in a vast majority of LAD-associated genes. However, confusingly, the authors concluded that LADs contribute to adipose lineage identity. This conclusion could be misleading. Furthermore, to make this conclusion, an experiment demonstrating the requirement of LADs or lamins for adipose stem cell differentiation must be presented. Alternatively, this main conclusion should be revisited.

Finding different chromatin features associated with expression of genes in LADs is the novel aspect of this paper, and the observations are interesting. However, the relationship between these different factors and the contribution of these different factors to gene expression (e.g. which factors are most and least correlated?) are not addressed. With this omission, the main message of this paper remains unclear.

A uniqueness of this paper is the use of adipogenic stem cell differentiation. Given that some laminopathies present lipodystrophy, the focus on this system is significant and interesting. However, there is almost no description about the biological aspect of adipogenic differentiation. This omission makes interpretation of LAD dynamics with respect to adipose lineage differentiation and identity almost impossible.

Despite these weaknesses, I still feel that this manuscript has a potential to contribute to the lamin field and can become a candidate for publication in Genome Biology, if extensively revised.

Specific comments:

Introduction: I urge the authors to include a paragraph describing the biological aspects of adipose stem cell differentiation. What differentiation potential does this stem cell population have? What is the relationship between in vivo and in vitro differentiation? What biological changes take place at what time points? Are there different differentiation methods and if so, how comparable is the author's method to others? These are some of the foundational questions to be addressed in Introduction.

Introduction: The 3rd paragraph "In most studies, vLADs have been reported as a single category,…"

This is an important point, and the authors indeed distinguished LAD gains and losses from LAD edge changes early on in the manuscript. However, features unique to edge changes were not defined. Is it because edge changes are not different from gains/losses of LADs? I would like to see more analyses focusing on these features.

Fig. 2d: Does the permutation take the sequence context, gene density, and feature size into account? A null hypothesis that LADs can occur anywhere in the genome is inappropriate in my opinion and requires strong justification. More appropriate background would be the B compartment or the union of all known LAD locations.

Fig. 3: The analyses presented in this figure are difficult to follow, due to the multiple categorical comparisons. Also, it is difficult to ascertain the robustness and the reproducibility of the dynamics of LADs. How are these changes affected by the sensitivity of LAD calls? How reproducible are these changes between biological replicates? What is the significance cutoff for the LAD changes? Finally, please show heatmaps and data tracks (not LAD calls) that show the dynamics of LADs.

Fig. 3c: The statistical significance of the difference of the Jaccard index is unclear and should be assessed using data from individual replicates.

Fig. 4: What is the relationship between GO enrichment and gene expression? For example, are genes linked to adipogenic GO terms in T0-T24-Lost LADs upregulated in this timing? It is difficult to understand the significance of the Fig. 4 data without the knowledge about the expression dynamics of the genes.

Fig. 4: Genes in Lost LAD > LAD class (Class i) are over-represented for GO terms related to non-adipogenic lineages. This is interesting observation. What is the biological significance of losing lamina association of these non-adipogenic genes between T0 and T24? I don't see any analysis that directly addresses the relationship between To-T24 LAD changes and T0-T24 gene expression changes. This is an important omission. Please include.

Page 6: "genes involved in brown adipogenesis are localized in…"

Does the differentiation system employed generate only white adipocytes and exclude brown adipocytes during the differentiation? More introduction about the differentiation system is needed, as stated above.

Page 6: "One should however mention that genes pertaining to adipogenesis are in vast majority already in i-LAD parts of the genome in undifferentiated ASCs… [16, 28]"

This seems an important point, but the data are not presented. The cited references do not appear to study LADs. Please show the data.

Page 7: "GO analysis highlights upregulation of genes involved in stimulus detection…"

It is unclear that which time point comparison are described in this sentence. This GO term analysis should be included in the main figure, so that readers can understand biological changes that take place during the differentiation. I suggest that Fig. 5a and b and associated GO analysis be presented at the beginning of this manuscript. A basic understanding of the differentiation process is necessary to understand this paper, especially for the general audience.

Fig. 5b: Based on this data, most gene expression changes take place within the first 4 hours of differentiation. Are these changes related to LAD changes within the first 4 hours?

Fig. 5d-e: One important finding of this manuscript is identification of discordant genes. What is the fraction of "discordant" genes among all LAD genes?

Fig. 5d-e: Can the apparent discordance be due to a local absence of lamina association? For example, we reported in Ikegami et al. PMID 21176223 that small gaps within large LADs harbor active genes. These small gaps might be "filled" in a LAD calling algorithm that favors large LADs. The actual lamina-association signals around the TSS of the LAD genes (concordant and non-concordant) should be analyzed (analysis similar to Fig. 7).

Fig. 7a: Please show a zoomed-in version of this data to distinguish between reduction vs. loss of LMNB1 association.

Page 10: "Note that we postulate that proximity of many of the inferred H3K27ac enhancers to their cognate genes implies…"

I don't understand this statement. Which LAD classes is this statement referring to? An earlier sentence stated that co-segregation is a rare event. This paragraph mentions proximity between K27ac sites and LAD genes several times, but there is no data measuring the proximity. What is the relationship between the distance and the location of K27ac sites within LADs vs outside?

Discussion: "We report evidence of radial reorganization of the ASC genome in the first hours of adipogenic differentiation, …"

This statement is misleading because it sounds as if lamina association was measured in the first hours, while the first time point of LAD measurement after T0 was T24.

Discussion: "The differential LAD/i-LAD outcome demonstrated here reflects a lineage-specific reorganization of peripheral chromatin"

This is an overstatement in my opinion as lineage specificity is not tested in this manuscript. To examine lineage specificity, different lineages need to be compared.

Discussion: "…suggesting vLADs play a role in adipogenic cell type-specific gene expression."

This statement is confusing because the data presented instead supported the idea that local chromatin context, not LADs, plays a major role in gene expression changes during adipogenic differentiation. A link between adipogenesis-specific gene expression and vLADs is missing or not clearly presented in the manuscript. This is related to my comment on Fig. 4.

Discussion: "Thus, vLADs identified here may contribute to the establishment and maintenance of adipose cell type identity…"

The data presented are insufficient to support this conclusion. To what extent do vLADs explain adipogenic gene expression changes? Are there any adipogenic linege-determining genes that show dynamic LAD association? Is there any data to support that lamin depletion compromises adipogenic differentiation? Data addressing these questions will be required to claim this conclusion.

Conclusion: "LADs constitute non-random features of adipose cell nuclear architecture contributing to the reinforcement of adipocyte lineage and cell type identity"

This statement needs to be rephrased as the data did not test for LADs' contribution to adipocyte lineage specification and identity.

Throughout the manuscript, at times, the authors appear to use the term "radial chromatin arrangement" to mean chromosome organization. In my opinion, radial chromatin arrangement has a specific meaning that is the position of chromatin along the radius of the nucleus. This study does not measure such physical properties. How LAD changes are related to actual physical locations within the nucleus remains unclear. This topic is particularly relevant to changes at LAD edges. So, I urge that the authors rephrase this term according to the context.

The use of "in-LAD", "i-LAD", and "non-LAD" is confusing. Can you use either in-LAD/non-LAD or i-LAD/LAD? Or, can you spell out, like "in LADs", "inter-LADs", "not in LADs" etc?

**Kohta Ikegami reviewed this manuscript. Review assignment accepted November 12, 2021; completed November 25, 2021. It is my standard policy to sign all my review reports, regardless of my recommendations. All correspondence should go via the Editor. Please do not remove this note.**

**Authors’ response**

Reviewer 1

1)We (BvS and a lab member) independently found the manuscript very dry and difficult to read. It provides some interesting points regarding the enhancers or the LAD dynamics, with the caveat that the enhancer-gene links are imputed. But overall, the reported findings seem incremental, strictly descriptive, and of limited novelty. The datasets may be useful for the community, but the most interesting ChIP-seq maps of lamina interactions were already published previously.

Considering these comments, we believe we have improved the paper in several ways. The text has been streamlined, better highlighting the main points. We have reorganized the paper (see also reviewer 2 on highlighting the biology) by first describing transcriptomic changes during adipogenesis (**Fig. 1**), then move to LADs (**Fig. 2**) to more clearly relate changes in LADs (and GO terms within) to expression changes (see also reviewer 2) (**Fig. 3g**, **Fig. 5a-c**). We now describe the 10 LAD classes only when it is most relevant and significantly adds to our understanding of LAD positioning; this means we also only describe some of the LAD classes (e.g. **Fig. 3**, dedicated to the 0-24 h LAD transitions; **Fig. 4c; Fig. 6a,e, Fig. 8c**), or highlight cLADs more clearly (**Fig. 5**). We now also clarify the enhancer relationship to DE genes in LADs by reorganizing the data (**Fig. 8c**) after a refinement of enhancer definition (doubleelite subset of GeneHancer; **Fig. 8a**). As described below, we also provide a more detailed and quantitative analysis of our LMNB1 ChIP datasets (**Fig. 2 and Fig. S3**). Regarding novelty, we would like to emphasize that (i) the lamin B1 enrichment (LAD) data have been completely reanalyzed, (ii) we provide an unprecedented, for human adipose progenitors, complete transcriptomic profile of early adipogenesis, every 4 h from 0 to 24 h, and (iii) we provide a unique histone modification dataset with the same 4-h time resolution.

2)Often the text is confusing and we had to read several paragraphs multiple times to understand them. It was often difficult to follow the logic of the experiments/analyses and the underlying rationale.

We are grateful for this comment and believe we have significantly improved the flow of the manuscript by focusing on the main findings and anchoring our genome data more strongly to the biology of adipose differentiation (see Reviewer 2 and Editor’s comment); see Results related to Fig, 1, 3, 4, 5, 6.

3)One reason why the results are difficult to digest is the choice of the authors to categorize LAD changes in 10 different categories spanning three different time points. Although we recognize the need to properly call LAD changes one could question if this division is really necessary throughout the manuscript, especially considering that gene expression and enhancer position do not strictly depend on any of these categories. Could some of the main findings of the manuscript not be described by simply calculating an LB1 score per gene or enhancer?

We agree with this view and have remedied to that in relevant sections to focus on the main findings:

we dedicate Fig. 3 to the five LAD categories identified between 0 and 24 h (Fig. 3a-g; see also Fig. 4c); we highlight GO terms for a subset of LAD classes more relevant to adipose biology (Fig. 4a) and describe expression changes in this subset (Fig. 4b); we also present expression data and histone PTM changes in a subset of these LAD classes (Fig. 6 and 7). When pertinent, data on other LAD classes are shown in Suppl. Figures.

Please note however that a main point in our paper is to distinguish changes in stand-alone LADs vs. LAD edges; this distinction clarifies data (Fig. 4b, d-f; Fig. 7a and Fig S7) which otherwise would not be informative. All in all however, we are very happy with this reviewer’s comment, which compelled us to substantially edit the manuscript.

4a)We have some concerns about the quality of LB1-ChIP seq data and the application of the EDD algorithm. Please show original ChIP-seq tracks of LB1 in Figure 1a, instead of binary LAD tracks only.

We now provide tracks as Log2(ChIP/input) ratios in Fig. 2a and Fig. S3a). We also show the called LADs for each replicate (Fig 2a, Fig S3a), which gave the basis for considering the union of the replicate LADs as final LAD sets considered in this paper. We now provide stronger evidence justifying the LADs considered thereafter.

4b)Please also provide an estimate of the quality of the ChIP tracks (signal to noise ratio, dynamic range, correlation between replicates). This is important because LAD borders and vLADs are usually marked by low level of LB1 and its definition by an algorithm is subjected to fluctuation because of noise.

We now provide 1) Pearson correlations of Log2(ChIP/input) ratios in 100-kb bins across the genome, 2) Pearson correlations of LMNB1 ChIP read counts from bam files in called LADs, between replicates (Fig. 2b), and 3) the dynamic range of LMNB1 ChIP enrichments as Log2(ChIP/input) in LADs (Fig S3b).

5)The only way to minimize this is to increase the number of replicates, while in this study only two reps are present. Some of these replicates yield very different numbers, like T72 rep1 vs rep2 where the number of LADs and the size of LADs is extremely different between replicates. This is even more complicated by the fact that the authors perform the EDD calling on the union of the two replicates. This suggest that gain and losses (for border or stand-alone LADs) can basically derive from one single replicate. More replicates should be added or at least intersection analysis should be performed. Also, the authors show only one track for LB1 chip and it does not seem of the highest quality.

We appreciate this concern. As outlined above, we now provide QC data on our lamin ChIPseq biological replicates, which we believe argue for using merged LADs within timepoints as final LAD calls. LMNB1 ChIP/input ratios (Fig. 2a and S3a) and Pearson correlations (Fig. 2b) show similarity in enrichment levels between replicates to justify calling LADs for each and considering the union as final calls. Ratios also show variations in enrichment levels between replicates, but profiles remain similar. These variations expectedly lead to some variability in EDD calls between replicates; but considering the high similarity of ratios between replicates it made sense to take the union of LADs between replicates as final LADs (Fig. 2c). We have also tested replicate intersects (not shown), but resulting LADs did not faithfully reflect all actual enriched domains, and logically biased towards cLADs.

Minor points:

1)The discussion could be further developed by a deeper interpretation of the data. For example: The dynamics at t24 (transient lamina association) are interesting (transient LADs vs transient iLAD) and could be discussed further. What would be the function of this transient repositioning to the lamina if it cannot be connected to gene expression and only marginally to enhancers?

We now address this issue in the Discussion.

2)In line 8 page 8 the authors say "Resilience of DE genes to changes in LAD status and to the overall

heterochromatic environment of cLADs. To claim that the authors should provide a quantitative analysis of H3K9me3 during differentiation

We now provide a quantification of H3K9me3 enrichment in LADs and i-LADs at each timepoint (Fig. 2e), in the 0-24 h LAD categories (gained/lost LADs, gained/lost edges, cLADs and i-LADs; Fig. 3e), and in active vs inactive sub-domains of cLADs (Fig. 6c) together with LMNB1 enrichment in these regions (Fig. 6d). We have also accordingly rephrased the sentence and section in question (p7).

3)In legend of figure 5 the letter "a" is repeated twice and "RNA-seq profiling of early adipogenic

differentiation" is either misplaced or absent from the figure.

This is fixed. Note that Figure number is changed.

4)Boxplot and violin plot in fig1C are not aligned.

This plot has now in fact been removed.

Reviewer 2

....One important and interesting finding is that adipogenic gene expression changes are correlated with genomic features other than lamina association in a vast majority of LAD-associated genes. However, confusingly, the authors concluded that LADs contribute to adipose lineage identity. This conclusion could be misleading. Furthermore, to make this conclusion, an experiment demonstrating the requirement of LADs or lamins for adipose stem cell differentiation must be presented. Alternatively, this main conclusion should be revisited.

We agree and have toned down some of our conclusions on this basis; please see below for details.

Finding different chromatin features associated with expression of genes in LADs is the novel aspect of this paper, and the observations are interesting. However, the relationship between these different factors and the contribution of these different factors to gene expression (e.g. which factors are most and least correlated?) are not addressed. With this omission, the main message remains unclear.

Point taken. As described below we have now taken this into account.

A uniqueness of this paper is the use of adipogenic stem cell differentiation. Given that some laminopathies present lipodystrophy, the focus on this system is significant and interesting. However, there is almost no description about the biological aspect of adipogenic differentiation. This omission makes interpretation of LAD dynamics with respect to adipose lineage differentiation and identity almost impossible.

We definitely agree. As detailed below, we have significantly improved the links between our genomic findings (LADs, RNAseq, GO terms, histone marks) and our biological adipose system.

Despite these weaknesses, I still feel that this manuscript has a potential to contribute to the lamin field and can become a candidate for publication in Genome Biology, if extensively revised.

We appreciate the reviewer’s positive view of the potential our manuscript presents, and argue below that we have now significantly improved it to meet the reviewer’s requirements and suggestions.

**Specific comments:**

Introduction: I urge the authors to include a paragraph describing the biological aspects of adipose stem cell differentiation. What differentiation potential does this stem cell population have? What is the relationship between in vivo and in vitro differentiation? What biological changes take place at what time points? Are there different differentiation methods and if so, how comparable is the author's method to others? These are some of the foundational questions to be addressed in Introduction.

We greatly appreciate this comment. The introduction has been revised accordingly by adding a new section on adipose stem cells (ASCs) and adipogenic differentiation (**Introduction**). Note however that transcriptional, chromatin, protein and phenotypic processes of adipogenesis have been extensively reported, so we do not provide details here (but instead, key Refs). We agree that throughout the paper (see below), a closer link between LADs, expression changes and adipocyte differentiation was needed; we have remedied to this.

Introduction: The 3rd paragraph "In most studies, vLADs have been reported as a single category,…" This is an important point, and the authors indeed distinguished LAD gains and losses from LAD edge changes early on in the manuscript. However, features unique to edge changes were not defined. Is it because edge changes are not different from gains/losses of LADs? I would like to see more analyses focusing on these features.

We apologize if features of LAD edges have not appeared clearly, and thank the reviewer for pointing this out. We did describe edge features in our initial manuscript, but we have now streamlined it (as requested by Reviewer 1) so descriptions of LAD edges in relation to other LAD classes are clearer. Note that histone PTM data in LAD edges are in dedicated **Fig. S7**. Edge features are reported throughout the manuscript, in **Fig 3b-g** (including new analyses such as LMNB1 levels, H3K9me3 levels, browser views and expression changes), **Fig. 4b,c,e,f** (overlaps with LADs in T72 and in proliferating cells), **Fig. 5a,b** and **Fig. S4a,b** (GO terms and related gene expression), and in **Fig. 5c,d** (browser examples).

Fig. 2d: Does the permutation take the sequence context, gene density, and feature size into account? A null hypothesis that LADs can occur anywhere in the genome is inappropriate in my opinion and requires strong justification. More appropriate background would be the B compartment or the union of all known LAD locations.

We apologize for failing to mention this: indeed, we did use as background the union of all known LAD locations on the same respective chromosomes in the target cell type. This is now done in the Methods under “Statistical tests...” and in Fig legends (**Fig. 4c,e)**.

Fig. 3: The analyses presented in this figure are difficult to follow, due to the multiple categorical comparisons. Also, it is difficult to ascertain the robustness and the reproducibility of the dynamics of LADs. How are these changes affected by the sensitivity of LAD calls? How reproducible are these changes between biological replicates? What is the significance cutoff for the LAD changes? Finally, please show heatmaps and data tracks (not LAD calls) that show the dynamics of LADs.

We appreciate these comments and have addressed them in several ways. 1) We provide a detailed analysis of LMNB1 enrichments in each replicate, quality control data (IGV tracks and Pearson correlations, dynamic range) and dedicate **Fig. 2 and Fig. S3** to this (see also **Fig. 6e,f** and comments below). Our data show reproducibility between our biological replicates. The data in former Fig 3 are now in **Fig 4**, which is now split up for clarity: **Fig 4c** only shows O/E ratios of overlap of the five T00-T24 LAD categories with LADs in T72 cells; **Fig. 4e** only shows O/E ratios of overlaps with LADs in proliferating cells, and **Fig. 4f** (now separate from Fig. 4e) focuses on percent overlaps. We believe breaking down these data make the story a lot easier to follow and understand.

Fig. 3c: The statistical significance of the difference of the Jaccard index is unclear and should be assessed using data from individual replicates.

We agree and have removed this dataset because it was not useful. We instead report a detailed analysis of LMNB1 ChIP data, as described in the previous point and in response to Reviewer 1, point

5.

Fig. 4: What is the relationship between GO enrichment and gene expression? For example, are genes linked to adipogenic GO terms in T0-T24-Lost LADs upregulated in this timing? It is difficult to understand the significance of Fig. 4 without the knowledge of the expression dynamics of the genes.

We appreciate the questions and now address this: **Fig. 5a,b** and **Fig. S4a,b** directly relate GO terms enriched in lost LADs and edges in particular (since these contain adipose-relevant genes) to changes in gene expression. This has made the paper more biologically relevant, in line with the reviewer’s (and the Editor’s) general comments. We are grateful for this.

Fig. 4: Genes in Lost LAD > LAD class (Class i) are over-represented for GO terms related to nonadipogenic lineages. This is interesting observation. What is the biological significance of losing lamina association of these non-adipogenic genes between T0 and T24? I don't see any analysis that directly addresses the relationship between To-T24 LAD changes and T0-T24 gene expression changes. This is an important omission. Please include.

This is again a valid point, related to the previous point, which we now specifically address in **Fig. 5a,b** (see above): Lost LADs are associated with higher expression levels, and re-gain of LADs (Lost LAD > LAD), with lower expression. This is also addressed in **Fig. 3g**, where we show by calculating Log2(fold expression changes) that gains and losses of LADs between 0 and 24 h coincide with respectively reduced and increased gene expression in these domains.

Page 6: "genes involved in brown adipogenesis are localized in…". Does the differentiation system employed generate only white adipocytes and exclude brown adipocytes during the differentiation? More introduction about the differentiation system is needed, as stated above.

Our differentiation system is a white adipogenic system which does not elicit expression of the key beige/brown adipocyte marker PRDM16. Eliciting beige adipogenic differentiation requires another protocol that includes the PPARG agonist rosiglitazone (such protocols have been published). We do not exclude however that some beige adipocyte markers may be elicited at some timepoint during in vitro white adipogenesis, but this is not the subject of this paper. We note that another beige/brown adipocyte marker, BNIP3, is down-regulated during differentiation. Both these genes are illustrated in **Fig. 5c**. As mentioned above, the Introduction now contains a section on adipose differentiation.

Page 6: "One should however mention that genes pertaining to adipogenesis are in majority already in i- LAD parts of the genome in undifferentiated ASCs… [16, 28]". This seems an important point, but the data are not presented. The references do not appear to study LADs. Please show the data.

We apologize for the reference mixup; this is now corrected. We also show the proportion of adipogenic genes, from the GSEA Hallmark_Adipogenesis in LADs vs. non-LADs in **Fig. 5d**.

Page 7: "GO analysis highlights upregulation of genes involved in stimulus detection…" It is unclear that which time point comparison are described in this sentence. This GO term analysis should be included in the main figure, so that readers can understand biological changes that take place during the differentiation. I suggest that Fig. 5a and b and associated GO analysis be presented at the beginning of this manuscript. A basic understanding of the differentiation process is necessary to understand this paper, especially for the general audience.

We agree and are thankful for this comment. We have moved gene expression data (former Fig. 5) to **Fig. 1** (to first focus on the biology of adipogenesis with our 4-h time resolution RNAseq data). We also combine gene expression clusters with GO term enrichment analysis (**Fig. 1e,f**) to provide a clearer understanding of biological changes taking place in the first 4-24 h of differentiation. We also as suggested provide a more detailed description relating LADs, GO terms, expression changes and time course.

Fig. 5b: Based on this data, most gene expression changes take place within the first 4 hours of differentiation. Are these changes related to LAD changes within the first 4 hours?

This is a good question by we have not addressed this directly. We find that in cLADs that show significant expression changes at 4-8 h (**Fig. 6a**), the low LMNB1 levels at these active sites, do not vary between T00 and T24. This suggests that these expression changes occur at sites of low lamin occupancy from the start (at T00). We cannot exclude however that some of the expression changes between T00 and T04 or T08 are not accompanied by changes in lamin association (in vLADs), but we have not examined this at this stage.

Fig. 5d-e: One important finding of this manuscript is identification of discordant genes. What is the fraction of "discordant" genes among all LAD genes?

We have determined the proportion of discordant genes – which we restrict to DE genes in cLADs – to be **11%** (72/653; **Fig. 5g**). We find expressed genes and DE genes in all LAD classes (**Table S2**); but since expression patterns are complex in all LAD classes, including vLADs which “come and go”, we find it more relevant, and clearer, to restrict a definition of “discordance” to genes in cLADs (note also that all expressed genes identified here in cLADs are also differentially expressed [DE]; **Table S2**).

Fig. 5d-e: Can the apparent discordance be due to a local absence of lamina association? For example, we reported in Ikegami et al. PMID 21176223 that small gaps within large LADs harbor active genes. These small gaps might be "filled" in a LAD calling algorithm that favors large LADs. The actual laminaassociation signals around the TSS of the LAD genes (concordant and non-concordant) should be analyzed (analysis similar to Fig. 7).

This is correct; we now provide evidence of reduced or abrogated LMNB1 association in regions of active genes (with a focus on cLAD genes); please see also the comment below and our new data pertaining to this point (**Fig. 6b-e**). As we have discussed earlier (Briand and Collas 2020 Genome Biol), and as pointed out here, we re-address the point of LAD call vs. lamin enrichment (**Fig. 2**). We now cite Ikegami PMID 21176223 which indeed is relevant here and was a regrettable omission from our part.

Fig. 7a: Please show a zoomed-in version of this data to distinguish between reduction vs. loss of LMNB1 association.

We now provide a new figure (**Fig. 6**) with a more detailed analysis of the reduction/loss of LMNB1 levels in cLAD sub-domains containing active genes (**Fig. 6a**) and corresponding histone PTMs (**Fig. 6b,d**). We also show that these cLAD sub-domains also display reduced H3K9me3, reflecting a local loss of heterochromatin (**Fig. 6c**). We show two more adipose-relevant loci (PXDN and INSIG1; **Fig. 6e**), aiming again to link our genome findings to adipogenesis, and as requested, zoom-in on the promoters of these genes (along with TRNT1), showing absence of LMNB1 around TSSs (**Fig. 6f**). With the quantification provided, we believe our claims are now well substantiated.

Page 10: "Note that we postulate that proximity of many of the inferred H3K27ac enhancers to their cognate genes implies…". I don't understand this statement. Which LAD classes is this statement referring to? An earlier sentence stated that co-segregation is a rare event. This paragraph mentions proximity between K27ac sites and LAD genes several times, but there is no data measuring the proximity. What is the relationship between distance and location of K27ac sites within LADs vs outside?

We apologize for the unclear statements. We have reworked this section with a more stringent definition of enhancers, using the GeneHancer double-elite subset (**Fig. 8a,b**), new data on enhancergene distances (**Fig. 8d,e**), and new (and clearer) data presentation on enhancer localization as a function of gene LAD class and time (**Fig. 8c**). The latter (**Fig. 8c**) also accommodates comments from Reviewer 1 point 3 on how confusing our manuscript was in some places. We hope the section on enhancers is now clearer and thereby more informative.

Discussion: "We report evidence of radial reorganization of the ASC genome in the first hours of adipogenic differentiation, …"

This statement is misleading because it sounds as if lamina association was measured in the first hours, while the first time point of LAD measurement after T0 was T24. We agree and have rephrased to “within the first 24 to 72 h of induction”; we have also removed the word “radial”, in light of other comments below.

Discussion: "The differential LAD/i-LAD outcome demonstrated here reflects a lineage-specific reorganization of peripheral chromatin"

This is an overstatement in my opinion as lineage specificity is not tested in this manuscript. To examine lineage specificity, different lineages need to be compared.

We agree and have rephrased this to “during early adipogenesis”.

Discussion: "…suggesting vLADs play a role in adipogenic cell type-specific gene expression."

This statement is confusing because the data presented instead supported the idea that local chromatin context, not LADs, plays a major role in gene expression changes during adipogenic differentiation. A link between adipogenesis-specific gene expression and vLADs is missing or not clearly presented in the manuscript. This is related to my comment on Fig. 4.

We agree and thank the reviewer for pointing this out. We have rephrase this to “...relieved from lamin interactions, to occupy a chromatin environment permissive for adipogenic gene expression”. According to this and other comments, we provide a more complete analysis of gene expression as a function of LAD dynamics in our system, and hope this is now clearer. We would like to add that chromatin context plays a major role in adipogenic gene expression: we now qualify this (as requested above) with observations that most adipogenic genes are already in i-LADs in undifferentiated ASCs (**Fig. 5d**).

Discussion: "Thus, vLADs identified here may contribute to the establishment and maintenance of adipose cell type identity…". The data presented are insufficient to support this conclusion. To what extent do vLADs explain adipogenic gene expression changes? Are there any adipogenic lineagedetermining genes that show dynamic LAD association? Is there any data to support that lamin depletion compromises adipogenic differentiation? Data addressing these questions will be required to claim this conclusion.

Re. the first point, we agree and have removed this statement to rephrase this paragraph.

Re. the following points, ee now better highlight the relationship between the vLAD localization of adipogenic genes and their expression (**Fig. 5b,c**) and expression of adipogenic-related genes in cLADs during differentiation (**Fig. 6e**). There is in fact evidence (not added to the Discussion) supporting the involvement of lamin A/C in adipogenesis, including lamin A/C mutation data in mouse (lipodystrophy model, Boguslavsky et al. 2006 HMG) and evidence of defective lamin interactions with, and impacts on expression of, adipogenesis-relevant genes (Oldenburg et al 2017 JCB). However there are to our knowledge no data directly assessing the effect of B-type lamin depletion on adipogenesis. Such studies would be very interesting. Since we do not have such data at hand, we have rephrased our discussion according to the reviewer’s comments.

Conclusion: "LADs constitute non-random features of adipose cell nuclear architecture contributing to the reinforcement of adipocyte lineage and cell type identity". This statement needs to be rephrased as the data did not test for LADs' contribution to adipocyte lineage specification and identity.

We agree and have rephrased (shortened) the conclusions accordingly.

Throughout the manuscript, at times, the authors appear to use the term "radial chromatin arrangement" to mean chromosome organization. In my opinion, radial chromatin arrangement has a specific meaning that is the position of chromatin along the radius of the nucleus. This study does not measure such physical properties. How LAD changes are related to actual physical locations within the nucleus remains unclear. This topic is particularly relevant to changes at LAD edges. So, I urge that the authors rephrase this term according to the context.

We agree and have accordingly removed the notion of “radial position” throughout the manuscript.

The use of "in-LAD", "i-LAD", and "non-LAD" is confusing. Can you use either in-LAD/non-LAD or i- LAD/LAD? Or, can you spell out, like "in LADs", "inter-LADs", "not in LADs" etc?

We apologize for the complexity. We now consistently refer to LADs or i-LADs (for inter-LADs).
